# Key triggers of adaptive genetic variability of sessile oak [Q. petraea (Matt.) Liebl.] from the Balkan refugia: outlier detection and association of SNP loci from ddRAD-seq data

**DOI:** 10.1038/s41437-023-00629-2

**Published:** 2023-06-14

**Authors:** Endre Gy. Tóth, Klára Cseke, Attila Benke, Botond B. Lados, Vladimir T. Tomov, Petar Zhelev, József D. Kámpel, Attila Borovics, Zoltán A. Köbölkuti

**Affiliations:** 1 Department of Forest Tree Breeding, Forest Research Institute (UOS-FRI), University of Sopron, Várkerület 30/A, Sárvár, 9600 Hungary; 2 Department of Landscape Architecture, Faculty of Ecology and Landscape Architecture, University of Forestry (UF), Kliment Ohridsky 10, Sofia, 1797 Bulgaria; 3 Department of Dendrology, Faculty of Forestry, University of Forestry (UF), Kliment Ohridsky 10, Sofia, 1797 Bulgaria; 4 Ottó Herman Environmental and Agricultural Technical School, Vocational School and College (Agricultural Vocational Centre of the Kisalföld Region), Ernuszt Kelemen 1, Szombathely, 9700 Hungary; 5 Departement of Applied Forest Genetics Research, Bavarian Office for Forest Genetics (AWG), Forstamtsplatz 1, Teisendorf, 83317 Germany

**Keywords:** Genetic variation, Genetic variation

## Abstract

Knowledge on the genetic composition of *Quercus petraea* in south-eastern Europe is limited despite the species’ significant role in the re-colonisation of Europe during the Holocene, and the diverse climate and physical geography of the region. Therefore, it is imperative to conduct research on adaptation in sessile oak to better understand its ecological significance in the region. While large sets of SNPs have been developed for the species, there is a continued need for smaller sets of SNPs that are highly informative about the possible adaptation to this varied landscape. By using double digest restriction site associated DNA sequencing data from our previous study, we mapped RAD-seq loci to the *Quercus robur* reference genome and identified a set of SNPs putatively related to drought stress-response. A total of 179 individuals from eighteen natural populations at sites covering heterogeneous climatic conditions in the southeastern natural distribution range of *Q. petraea* were genotyped. The detected highly polymorphic variant sites revealed three genetic clusters with a generally low level of genetic differentiation and balanced diversity among them but showed a north–southeast gradient. Selection tests showed nine outlier SNPs positioned in different functional regions. Genotype-environment association analysis of these markers yielded a total of 53 significant associations, explaining 2.4–16.6% of the total genetic variation. Our work exemplifies that adaptation to drought may be under natural selection in the examined *Q. petraea* populations.

## Introduction

Forest ecosystems are undergoing unprecedented changes in environmental conditions due to global change impacts (González de Andrés 2019; Pouresmaeily 2022; Pörtner et al. 2022). These changes involve the simultaneous and rapid alteration of several key environmental parameters that control the dynamics of forests (Thom and Seidl 2022). As a result, modifications in tree species’ dominance and distribution, productivity, and nutrient cycles are expected (Gea‐Izquierdo and Sánchez‐González 2022; Boonman et al. 2022; Elsen et al. 2022). It is important to determine how the life processes of trees will be affected by new specific competitive and climatic conditions, since large-scale, consistent monitoring of forest ecosystems plays a key role in preparing for the impact of extreme events (Kijowska-Oberc et al. 2020).

Economic evaluation based on the output of the large-scale scenario model EFISCEN grouped major European tree species according to their economic performance (https://efi.int/knowledge/models/efiscen). Among seven groups of tree species, *Quercus petraea* (Matt.) Liebl. was ranked fourth in terms of economic importance, being of considerable productivity (Hanewinkel et al. 2013). In addition to its economic importance, the species is highly valued in nature conservation terms (Mölder et al. 2019). In terms of adaptation, on one hand, sessile oak is widely regarded as a forest species with high adaptiveness (Kohler et al. 2020) since its drought tolerance and storm resistance are superior to those of other common trees (Kunz et al. 2018). On the other hand, the climate is shifting rapidly (Loarie et al. 2009) and due to the long lifespan of oaks, shifts in the genetic composition of populations are slow, opportunities for adaptation are limited, making the predictions about its response to climate change contradictory (Sáenz-Romero et al. 2017). Considering oaks adaptiveness, important works (Temunović et al. 2020; Lang et al. 2021; Guichoux et al. 2013) analyse potential candidate genes for stress responses. Available resources on functional drought candidate genes are considered the works of Homolka et al. (2013), Lepoittevin et al. (2015) and Rellstab et al. (2016). However, as future adaptedness requires beneficial alleles from habitats currently matching future climatic conditions, the importance of collecting more information about the Balkans in terms of adaptation of sessile oak has high priority in genetic research.

The genetic diversity of *Q. petraea* has been evaluated using different markers at different scales by numerous authors, including but not limited to the morphological research of Streiff et al. (1998), Bruschi et al. (2003), isoenzyme research of Zanetto and Kremer (1995) and Kremer and Zanetto (1997); microsatellite research of Muir et al. (2004); research using chloroplast genetic markers of Petit et al. (1993), Bordács et al. (2002), and Mátyás and Sperisen (2001); markers developed from EST databases (Lang et al 2021) or by using next generation sequencing of the whole genome (Leroy et al. 2020).

Reduced representation sequencing (Miller et al. 2007; Lewis et al. 2007; Baird et al. 2008) is a simple, cost-effective approach for generating large amounts of SNP data and is gaining popularity in species conservation (Fuentes‐Pardo and Ruzzante 2017) and phylogenetic studies (Hipp et al. 2018). Such genome-wide studies can also provide valuable information on adaptive gene flow, selection and speciation (Hipp et al. 2014, 2018; Cavender-Bares et al. 2015; Eaton et al. 2015; Fitz-Gibbon et al. 2017; Pham et al. 2017; Deng et al. 2018; Kim et al. 2018; Ortego et al. 2018; Jiang et al. 2019; Blanc-Jolivet et al. 2020; Degen et al. 2021).

As we mentioned in our previous work (Tóth et al. 2021), while sessile oak populations are well studied in Europe, the Central-Eastern European region, including the Balkan Peninsula, with the exception of some important works (Zanetto and Kremer 1995; Gömöry et al. 2001; Bordács et al. 2002; Slade et al. 2008; Neophytou et al. 2010) has been less investigated with high-resolution and genome-wide genetic markers. The genetic diversity in the Balkan region is generally high due to many refugia in this area harbouring differentiated genetic lineages and high environmental heterogeneity (Birks and Willis 2008; Gömöry et al. 2020). For this reason, the area is considered an important source of genetic material not only for forestry (Tzedakis 2004; Feliner 2011; Fassou et al. 2020) but also for research on the genomic architecture of adaptation.

Local adaptation occurs when individuals from a population have higher average fitness due to genetic changes in their local environment than individuals from other populations of the same species (Savolainen et al. 2013). Exploring this phenomenon, to identify the environmental factors responsible for genetic variation and gene variants that drive adaptation to the environment, are the most important aims. While some models predict that specific genes with large effects may be more important than other loci, other theoretical works emphasize the importance of polygenic traits in mediating local adaptation (Kawecki and Ebert 2004). However, to apply any of these approaches to elucidate the genetic variation underlying local adaptation, generation of large numbers of SNPs is required.

To address the need for optimal marker resolution, RAD sequencing (Peterson et al. 2012) fulfils the requirements for determining individual sequence genotypes that can be tuned to sample a wide range of randomly distributed regions genome-wide. Nevertheless, while the approach clearly permits genotyping of multiple individuals, it has its limit, namely, the ability to genotype for only the number and type of markers needed for the experiment. To overcome this deficiency, RAD sequencing data can be coupled with a reference genome or a study-specific annotated sequence database, which is advantageous for several reasons: improving the reliability of genotype calls (Torkamaneh et al. 2016), reducing the required coverage for accurate genotyping (Davey et al. 2011), providing a greater number of SNPs, improving downstream population genetic inferences (Shafer et al. 2017) and allowing SNP annotation with gene information (Gurgul et al. 2019). SNP annotation can identify the target genes of the analysis and to separate the functional vs. non-functional diversity of the genome, with high conservation implications (Johnson et al. 2018).

One of the major interests of current quantitative genetics is to explore the exact number, distribution and interaction of loci affecting the variations in adaptively important traits. The first step in this exploration process is SNP discovery. The second is to distinguish the molecular variation of the SNPs that are neutral from those that are subject to selection since selection is the predominant driver of differentiation in phenotypic traits (Darwin 1859; Rieseberg et al. 2002; Matesanz et al. 2010; Kremer and Hipp 2020). However, species have different sensitivity in terms of growth, survival and reproduction. Changes in environment are not always reflective on the selection signature and moreover, one SNP could have multiple associations with different environmental variables (Ahrens et al. 2018). Still, searching for loci under selection with important considerations regarding the underlying neutral genetic structure may provide valuable information on the adaptation to local conditions, as adaptation can cause subtle changes in allele frequencies (Rellstab et al. 2015). For this purpose, various specific strategies have been developed. These approaches include DNA-based, mRNA-DNA, protein-DNA, DNA-environment, and phenotype-based methods (Vasemägi and Primmer 2005). The *F*_ST_-based method is a DNA-based approach that uses multiple-population tests to estimate outliers (Beaumont 2005). During the identification of outlier loci by this method, it is necessary to use different approaches to minimize the false-positive rate since the validation of the detected outliers is highly important (Tsumura et al. 2012). However, even by different approaches, outlier tests make the assumption that selection pressures differ among populations and also do not link specific selection pressures that underlie adaptation (Rellstab et al. 2015). Additionally, identifying outlier loci is complicated by the occurrence of asymmetric introgression between oak species, which may be attributed to differences in colonization history and result in outliers retaining signatures of past introgression events (Bierne et al. 2011; Guichoux et al. 2013). For this reason, another way is needed to identify loci under selection, to see which of them are correlated with environmental gradients using allele distribution models (Joost et al. 2007; Holderegger et al. 2008; Manel and Segelbacher 2009). The basic hypothesis of allele distribution models is that natural selection due to heterogeneity generates changes in allele frequencies at loci linked to selected genes (Endler 1986; Hirao and Kudo 2004; Schmidt et al. 2008). The highly differentiated markers assessed by these methods become candidate genes for adaptation to the environmental factors in question, tracing the patterns of fundamental evolutionary processes: differential survival or reproduction of genetically based phenotypes in response to environmental challenges. However, environmental association analyses also have important limitations. Their main limit is that they might result in high rates of false positives (Frichot et al. 2015, Rellstab et al. 2015), requiring preliminary assessments of population structure to avoid false positive associations (Ahrens et al. 2018, Capblancq and Forester 2021). To avoid false positives, rather than contrasting association analyses and outlier detection methods, a more effective approach may be to combine them and link the positively identified SNPs to gene function using gene ontology analyses (Rellstab et al. 2015).

In this study, we identified and investigated the genetic variation at several stress-response loci in natural sessile oak populations from the Central-Eastern European region. The objectives of the study were (i) to reveal the genetic structure, diversity and differentiation of the sampled populations; (ii) to identify stress-response loci in our RAD sequencing dataset after mapping paired-end reads to the *Q. robur* reference genome; (iii) to explore the number and distribution of loci affecting the variations in the adaptively important traits by *F*_ST_ outlier detection and associations of SNP allele frequencies with environmental variation; and (iv) by annotating these loci, to describe the biological process, molecular function and cellular component, assuming the same function in both the resulted, already annotated model species, and *Q. petraea*.

## Materials and methods

### Plant materials

Sampling was designed to cover heterogeneous climatic conditions in the southeastern natural distribution range (CE-Europe) of *Q. petraea*, extending to Bulgaria, Hungary, Romania, Serbia, Bosnia and Herzegovina, Kosovo and Albania (Fig. S1). Altogether, 180 individuals from 18 natural populations were sampled in the framework of a former project, aiming to genotype a large number of samples and single-nucleotide polymorphisms (SNPs) from key geographic regions by RAD-seq (restriction-site associated DNA sequencing) (Table S1). Sampling was carried out using the classification system and taxonomic descriptors of Schwarz (1936). Detailed information on the sampling strategy can be found in Tóth et al. (2021).

### Genotyping

A variation of RAD sequencing, that is suitable for high-confidence genome-wide SNP loci discovery, double-digest RAD-seq (ddRAD-seq), was used to genotype all individuals (Peterson et al. 2012). The details of the wet-laboratory procedure, sequencing and raw sequence editing are described in Tóth et al. (2021). Key wet-laboratory steps are provided in the Supplementary Materials. Raw data have been deposited in the NCBI Sequence Read Archive (SRA); BioProject ID: PRJNA699096. The dataset can also be accessed at 10.5281/zenodo.3908963.

### Sequence editing and genome mapping

**Fig. 1:**
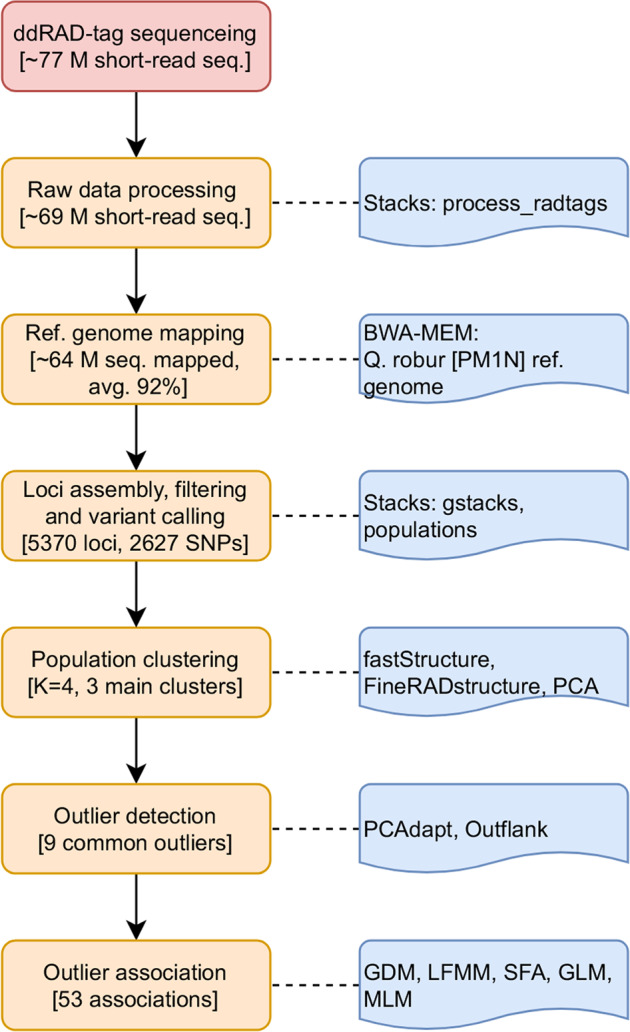
Schematic representation of the workflow used in this study. The main bioinformatic steps are visualised, including data processing, assembly, mapping, filtering, variant calling, reference mapping, diversity-differentiation analyses, outlier SNP detection and association.

Bioinformatics processing was carried out on a Silicon Computers (SGI) Unix-based HPC server, allocating 40 cores (80 threads) and 38 GB of RAM. Key steps of the bioinformatics pipeline are shown in Fig. 1.

Short read sequences (~77 M) were demultiplexed and adaptor-trimmed by using MiSeq Control Software (Illumina, San Diego, CA, USA). The resulting sequences (69,993,001) were 3′ and 5′ end-trimmed using the FastQ Toolkit. Reads with a mean quality score <30 and shorter than 200 bp were filtered. Computational processing of short-read data was carried out with Stacks 2.0 (Catchen et al. 2013; Rochette et al. 2019). Whole reads were quality filtered using a sliding-window method (15% of read length) implemented with ‘process_radtags’ (Rochette et al. 2019). Reads having a quality score below 90% (raw Phred score of 10) were discarded (Catchen et al. 2011). Using ‘process_radtags’, reads were truncated to 200 bp as a prerequisite for further processing and to avoid the lower-quality bases present at the ends of the reads (Catchen et al. 2011). During this filtering step, 42,273 sequences were discarded. In addition, one individual was removed from the dataset (BU2-10) since an insufficient number of high-quality reads remained after filtering.

Paired-end reads were mapped to the *Q. robur* reference genome (PM1N [haploid version]; http://www.oakgenome.fr; Plomion et al. 2018) using BWA-MEM v0.7.17 (Li 2013), which is designed for mapping reads (70 bp to 1 Mbp) against large reference genomes and has already been successfully used in oak studies (Fitz-Gibbon et al. 2017; Konar et al. 2017; Ramos et al. 2018). During mapping, parameters were set to default (Li 2013), and the unassigned scaffolds of the reference genome were excluded. Sequences of individual samples in SAM format were sorted with SAMtools v1.10 software (Li et al. 2009) and then converted to a BAM file. Reads with a mapping quality less than five were removed (MAPQ > = 5). The result of mapping was evaluated with the SAMtools ‘flagstat’ option and used for calculating the individual- and population-level summary statistics presented in Table S2, Table S3 and Fig. S2. The successfully mapped sequences (~64 M), later termed RAD loci, were kept for further downstream processing.

In Stacks, the ‘gstacks’ programme reconstructs loci and creates a SNP catalogue by incorporating paired-end reads that have been aligned to the reference genome using a sliding window algorithm (Catchen et al. 2013; Rochette et al. 2019). Unpaired reads were removed to avoid reads supporting a variant aligned to only one strand (strand-bias error). The ‘populations’ programme was used to call SNPs across the whole sample set. In this step, SNP markers with a minor allele frequency <0.05, a missing individual rate >0.8, and significant deviation from Hardy–Weinberg equilibrium (HWE, *p* < 1 × 10^−5^) were filtered out (Xiong et al. 2009; Marees et al. 2018). In addition, only a single SNP per locus was kept to have independent loci for later model-based statistical approaches. The ‘minimum number of populations’ parameter was set to 18 to identify loci that were present in all populations.

### Genetic structure and diversity

Population groups were estimated using three different methods, namely the Bayesian model-based clustering algorithm of fastStructure v 1.0 (Raj et al. 2014), fineRADstructure (Malinsky et al. 2018), and Principal Component Analysis (PCA).

fastStructure was run with default settings and 100-fold cross-validation on the 179 samples, testing for the best number of groups of populations (K) ranging from K = 2 to 9. The Python script ‘ChooseK’, included with the fastStructure package, was used to choose the number of groups that maximize the log-marginal likelihood lower bound (LLBO; Beal 2003) according to Raj et al. (2014). The mean population membership probability was manually calculated in MS Excel based on the Q-matrix values produced by fastStructure. fineRADstructure infers population structure via shared ancestry based on the autosomal loci, and focuses on the most recent coalescent events providing information on relatedness (estimates co-ancestry), which is informative in situations of contemporary gene flow. Individuals were assigned to populations using 1,000,000 iterations sampled every 1000 steps with a burn-in of 100,000. Estimated co-ancestry values were sorted according to populations and plotted as a heatmap. Principal component analysis (PCA) was performed using the ‘factoextra’ (Kassambara and Mundt 2017) and ‘FactoMineR‘ (Lê et al. 2008) packages in R (R Core Team 2013) to assess genetic differentiation among populations. The results were visualized using the ‘pophelper’ (Francis 2017) and ‘ggplot2’ (Wickham et al. 2016) packages and also plotted on a topographic map using ESRI ArcGIS (ArcMap 10.2.2, Redlands, CA, USA).

Expected heterozygosity (H_e_), observed heterozygosity (H_o_), and the inbreeding coefficient (*F*_IS_) were calculated for each population and for each genetic cluster in R using the ‘adegenet’ package (Jombart and Ahmed 2011). The significance of *F*_IS_ values was calculated separately with the ‘hierfstat’ package (Goudet 2005) using 1000 permutations. Allelic richness (Ar) was calculated using the ‘popgenreport’ package (Adamack and Gruber 2014). Private alleles (PAs) were counted in each population using the ‘poppr’ package (Kamvar et al. 2014). The diversity statistics are presented in Table S4. Genetic differentiation between populations and clusters was measured using the fixation index (*F*_ST_) (Nei 1973) by creating a pairwise distance matrix using the ‘hierfstat’ package and visualized as a heatmap (Fig. S3). In addition, an unweighted pair group method with arithmetic mean (UPGMA) dendrogram was created, with 1000 bootstrap resampling, based on the *F*_ST_ distances using the ‘ggdendro’ package in R (de Vries and Ripley 2013).

### *F*_ST_ outlier detection

Since the application of multiple selection tests (distinct approaches; e.g., *F*_ST_ frequency or Bayesian based) can outperform single tests in the detection of loci under directional selection, we applied distinct approaches to see if they converged and detected the same outliers (Tsumura et al. 2012; De La Torre et al. 2019). Altogether, two tests were performed: the PCA-based procedure implemented in PCAdapt 4.3.3 (Privé et al. 2020) and the *F*_ST_ frequency-based approach used by Outflank (Whitlock and Lotterhos 2015).

PCAdapt performs a PCA of a scaled genotype matrix and regresses all SNPs against the PCs to obtain a matrix of Z scores. Then, the robust Mahalanobis distances of these Z scores are computed to integrate all PCA dimensions into one multivariate distance for each SNP. Distances approximately follow a chi-squared distribution, thus enabling the calculation of a *p* value for each SNP (Privé et al. 2020). PCAdapt was found to be more powerful than former genome scans (Luu et al. 2017; Privé et al. 2020). Outflank identifies *F*_ST_ outliers, loci with atypical values of *F*_ST_, by inferring a distribution of neutral *F*_ST_ using likelihood on a trimmed distribution of *F*_ST_ values. In this analysis, the ‘number_of_samples’ parameter was set to 18 (a number equal to the populations sampled), the ‘LeftTrimFraction’ was set to 0.08, the ‘RightTrimFraction’ to 0.30, and the Hmin parameter was left at the default setting (0.1). The false discovery rate threshold for calculating q-values first was 0.05, as set by default (Whitlock and Lotterhos 2015), however in our final analysis a more stringent value of 0.01 was used.

The loci containing the significant SNPs was extracted from the initial dataset and annotated using the NCBI’s BLASTN and BLASTX services (https://www.ncbi.nlm.nih.gov/), as an example presented in Table S5. Finally, the type of nucleotide change, substitution type (synonymous or non-synonymous) and product change were determined by DnaSP (Rozas et al. 2017). The sequence of each locus can be accessed at 10.5281/zenodo.7763329.

### Outlier association

To test for associations of loci (SNPs) with environmental variables (GEA; genotype–environment associations), five different regression approaches were applied.

First, an environmental dataset of 84 monthly, seasonal, and annual variables was created by extracting climate data from WorldClim 1.4 (current: 1960–1990 based on De La Torre et al. 2019) in 30 arcsec-resolution (≤1 km) layers (Hijmans et al. 2005) and from ENVIREM 1.0 (current: 1960–1990) in 30 arcsec-resolution (≤1 km) layers (Title and Bemmels 2018) using the ‘raster’ (Hijmans and van Etten 2015), ‘rgeos’ (Bivand et al. 2017), and ‘rgdal’ (Bivand et al. 2015) R packages. The environmental variables were filtered for co-correlation between the environmental variables using Pearson’s correlation with the ‘caret’ package in R (Kuhn 2009), and by a step-by-step VIF (variance inflation factor) calculation, using a VIF threshold of 10, as implemented in the ‘usdm’ (Naimi 2017) and ‘fmsb’ (Nakazawa 2022) packages in R. Of the 84 variables, 14 variables were co-correlated with a *r*^2^ value below 0.75 and with a VIF < 10, thus were subsequently used for the association analyses. Selected environmental variables are detailed in Table S6.

Then, a matrix regression model (GDM) implemented in the ‘gdm’ package (Fitzpatrick et al. 2021), a latent factor mixed model (LFMM) implemented in the ‘LEA’ package in R (Frichot et al. 2013), a single-factor analysis of variance (SFA), a general linear model (GLM) (controlling for Q) and a mixed linear model (MLM) (controlling for Q + k) using TASSEL 5.2.73 (Bradbury et al. 2007) were performed.

GDM is a non-linear distance-based approach that models GEAs and adapts to a variable rate of change in allele frequencies along environmental gradients (Fitzpatrick and Keller 2015; Varas-Myrik et al. 2022). The method uses flexible splines (three as default) for fitting nonlinearity relationships between population dissimilarity and environmental variables as predictors (Ferrier et al. 2007). To investigate each outlier SNPs separately, we applied the protocol published in Fitzpatrick and Keller (2015) using the ‘gdm’ R package (Fitzpatrick et al. 2021). To measure population dissimilarities for each outlier SNP, locus-specific *F*_ST_ between all pairs of populations were calculated, as suggested by Fitzpatrick and Keller (2015), and using the ‘hierefstat’ R package. Best-fitted predictor’s spline present significant curvilinear relationships and indicates the total magnitude of change as a function and how the rate of change varies (Fitzpatrick and Keller 2015).

The LFMM approach uses a hierarchical Bayesian mixed model that accounts for covariation of alleles and the environment and for hidden population structure (via the K-value in the algorithm) while maintaining a relatively low false detection rate (de Villemereuil et al. 2014). For the analysis, our fastStructure-detected genetic clusters were chosen for the demographic background model. One hundred independent LFMM runs for each value of K with 10,000 iterations of the Gibbs sampling algorithm and a burn-in period of 5000 cycles were performed. |z | -scores for all loci were combined using the Fisher-Stouffer method (Brown 1975), and the resulting p values were adjusted using the genomic inflation factor (λ) (Devlin and Roeder 1999). A false discovery rate (FDR) correction of 1% was further used in *p* value adjustment using the ‘q-value’ package in R (Storey et al. 2021).

SFA, which does not consider population structure, was performed using each marker as the independent variable. Mean performance was compared between allelic classes using the general linear model (GLM), similarly to Kwon et al. (2013). The GLM (controlling for Q) and MLM (controlling for Q + k) analyses, for correcting population structure and relatedness, were performed using the Q-matrix (Q), which was obtained from the former fastStructure analysis, and a kinship (k) matrix which was calculated by the tool ‘Kinship’ with the Scald_IBS method built in TASSEL (Bradbury et al. 2007). We applied 1000 permutations for each test, and the p values of associated markers were tested against non-adjusted, Bonferroni-adjusted and FDR-adjusted significance thresholds at 0.05 (conservative), 0.01 and 0.001 (stringent) significance levels. Corrections of p values were carried out using the ‘dplyr’ (Mailund 2019) package in R.

## Results

### Bioinformatics data processing

Our pipeline yielded 64,378,333 short read sequences after filtering that were mapped onto the *Q. robur* reference genome with an average success rate of 92.43% (Table S2, Table S3 and Fig. S2). After processing, we reconstructed 5370 loci from an initial set of 410,445 loci, of which none had passed the filtering protocol (missing rate of sample/population and below the minor allele frequency (MAF) threshold). The final dataset consisted of 2,615,792 sites, in which we identified 2627 highly polymorphic variant sites (SNPs). The mean genotyped sites per locus was 474.24 base pairs (S. E. = 1.77). The Variant Call Format (VCF) file can be accessed at 10.5281/zenodo.7763329.

### Population structure and diversity

**Fig. 2:**
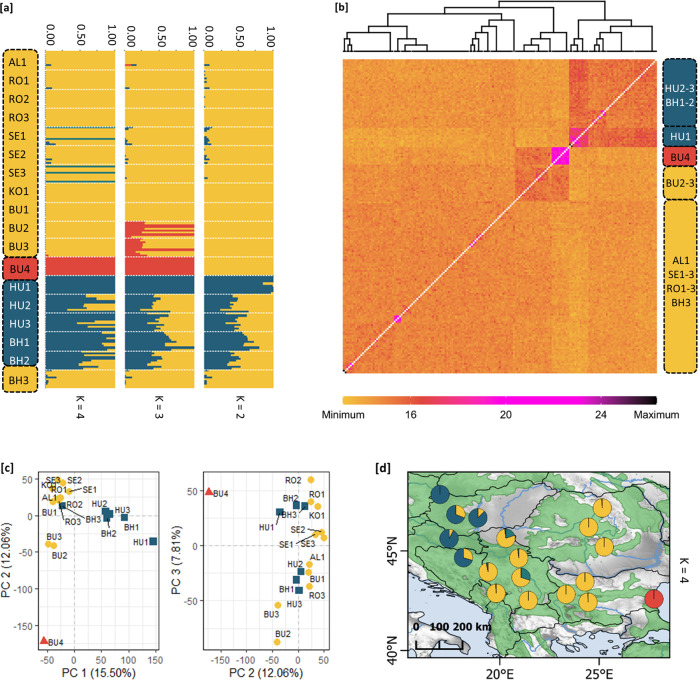
Estimates of genetic structure and differentiation. **a** Bayesian estimation of population structure in 18 populations of *Quercus petraea* using reference-mapped loci at K 2-4, as determined by fastStructure (Raj et al. 2014). The most likely number of clusters is defined by the ‘ChooseK’ method that maximizes the log-marginal likelihood lower bound (LLBO) according to Raj et al. (2014). **b** Estimated co-ancestry heatmap of fineRADstructure (Malinksy et al. 2018), individuals are ordered according to the fastStructure output. **c** Principal component analysis (PCA) of populations (PC1-PC2 and PC2-PC3 axes). **d** Geographic map of estimated population structure at K = 4 (mean population probabilities). Population abbreviations are as explained in Table S1.

The Bayesian clustering algorithm of fastStructure resulted in K = 4 as the number of groups that maximized the log-marginal likelihood lower bound (LLBO) for the SNP data. At this LLBO, we identified three main genetic clusters (Clusters 1, 2 and 3) and few introduced individuals as a separate group (Fig. 2; within SE1 and SE3 populations). While the populations of Cluster 1 (AL, RO, SE, KO, and BU1–3) and Cluster 3 (BU4) were clearly separated, the individuals of Cluster 2 (populations of HU, BH1 and BH2) were highly admixed (Fig. 2). This pattern was also evident on the co-ancestry heat map of fineRADstructure. BU4 represented close familial relationships as indicated by an island of high co-ancestry values. In addition, high values were detected at the admixed populations of HU, BH1 and BH2. PCA, by explaining 27.56% of the variance at PC1 vs. PC2 and 19.87% of the variance at PC2 vs. PC3, separated Cluster 1 and 2, as well as Cluster 3 (BU4) in both ordinations (Fig. 2). The geographic extent of detected clusters indicated a north–southeast gradient, along which the southernmost Mediterranean Cluster 3 (red) towards the west, Cluster 1 (yellow) became dominant in continental regions and Cluster 2 (blue) increased towards Central Europe (Fig. 2).

Genetic differentiation was generally low among populations (*F*_ST_ ranging from 0.003–0.119) and among the genetic clusters (*F*_ST_ ranging from 0.022–0.083). Cluster 3 showed the highest differentiation compared to Clusters 1 and 2 (*F*_ST_ = 0.065 and 0.083), while differentiation between Clusters 1 and 2 was much lower (*F*_ST_ = 0.022) (Fig. S3). The UPGMA dendrogram of Nei’s genetic distance showed the closest relationship (shortest genetic distance) between Cluster 1 and Cluster 2, while Cluster 3 was positioned on a separate branch with a much greater genetic distance (Fig. S3).

Moderate levels of genetic diversity were observed, and the values were balanced at the population level (H_e_ = 0.181–0.223; H_o_ = 0.166–0.210), as detailed in Table S4. Allelic richness values were also balanced (Ar = 1.570–1.746). Interestingly, BU4 showed the lowest diversity values (H_e_ = 0.181; H_o_ = 0.166; Ar = 1.570). Significant inbreeding depression was not detected (*p* < 0.05). Private alleles were absent, and only HU3 had a unique allele. Diversity among genetic clusters was similar; only Cluster 3, which contained only the BU4 population, showed slightly lower diversity values (Table S4).

### *F*_ST_ outlier detection

**Fig. 3:**
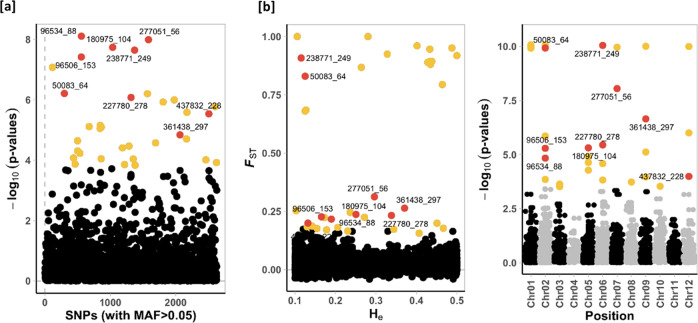
Detection of loci being under selection (outlier SNPs). Loci marked with yellow are significant for the specific approach, and loci with red are common among different approaches, as indicated in Table 1, and thus considered to be robust outliers. **a** The PCA-based method implemented in PCAdapt by Luu et al. (2017), and **b** the *F*_ST_ frequency-based method by Outflank (Whitlock and Lotterhos 2015).

Outlier detection methods revealed different numbers of SNPs as being under selection with different levels of significance. PCAdapt identified 34 SNPs, while Outflank identified 38 SNPs. In both approaches, thresholds were highly stringent with q-values < 0.01 and *p* ≤ 0.001, respectively. However, we considered robust outliers to be the SNPs detected jointly with the two different approaches. In this way, nine SNPs (0.34%) located at different loci, and at six different chromosomes (Chr. 2: 50083_64, 96506_153, 96534_88; Chr. 5: 180975_104; Chr. 6: 227780_278, 238771_249; Chr. 7: 277051_56; Chr. 7: 277051_56; Chr. 9: 361438_297; Chr. 12: 437832_228) were considered robust outliers (Table 1, Fig. 3). Those SNPs that were presented in NCBI databases resided in four different functional regions. Two SNPs found in titin homologues, one in bHLH162 transcription factor, one in hydroxymethylglutaryl-CoA synthase coding region, and one in a hypothetical protein coding region (possible double-strand damage repair) (Table 2). Functional regions with known CDS parts (96506_153, 96534_88, 277051_56) consisted three non-synonymous substitution (Table 2).

**Table 1:** Summary of outlier SNPs detected jointly by PCAdapt (Luu et al. 2017) and Outflank (Whitlock and Lotterhos 2015).

	PCAdapt			Outflank			
SNP	*p*-value	*q*-value	-Log10(*p*-value)	He	FST	*q*-value	*p*-value
50083_64	6.110E-07	1.665E-04	6.214E + 00	0.125	0.830	0.000E + 00	0.000E + 00
96506_153	3.770E-08	1.620E-05	7.423E + 00	0.165	0.227	2.584E-04	5.086E-06
96534_88	7.720E-09	1.080E-05	8.113E + 00	0.190	0.217	6.549E-04	1.450E-05
180975_104	1.810E-08	1.200E-05	7.742E + 00	0.251	0.237	2.574E-04	4.855E-06
227780_278	8.270E-07	1.969E-04	6.083E + 00	0.338	0.233	1.968E-04	3.551E-06
238771_249	2.240E-08	1.200E-05	7.650E + 00	0.115	0.908	0.000E + 00	0.000E + 00
277051_56	1.010E-08	1.080E-05	7.997E + 00	0.296	0.313	5.945E-07	8.775E-09
361438_297	1.440E-05	1.618E-03	4.843E + 00	0.371	0.264	1.427E-05	2.224E-07
437832_228	2.880E-06	4.415E-04	5.540E + 00	0.132	0.200	3.994E-03	1.015E-04

**Table 2:** Functional annotation and characteristics of outlier SNPs detected as being under selection.

ID	Chrom.	Position	SNP (ref./alt.)	Subs. Type^a^	Codon change	Product change	Function (BLASTn/BLASTx)
50083_64	Chr02	14386439	T/G	unk	unk	unk	hypothetical protein CFP56_76761 [*Quercus suber*] (DNA damage repair in Arabidopsis TAIR DB)
96506_153	Chr02	82808405	G/T	NS	ACG/AAG	Thr/Lys	titin homologue (LOC115976593) [*Quercus lobata*]
96534_88	Chr02	82833650	T/C	NS	AAC/GAC	Asn/Asp	titin homologue (LOC115976593) [*Quercus lobata*]
180975_104	Chr05	7935674	C/T	unk	unk	unk	transcription factor bHLH162 (LOC126725984) [*Quercus robur*]
227780_278	Chr06	15796585	A/T	unk	unk	unk	ns
238771_249	Chr06	32822044	T/C	unk	unk	unk	ns
277051_56	Chr07	33237883	T/C	NS	ATG/ACG	Met/Thr	ns
361438_297	Chr09	40682604	G/A	unk	unk	unk	ns
437832_228	Chr12	13562544	G/A	unk	unk	unk	hydroxymethylglutaryl-CoA synthase (LOC115971599) [*Quercus lobata*]

^a^substitution type, *S* synonymous, *NS* non-synonymous, *unk* unknown.

*ns* no significant similarity (or uncharacterized transcript variant/protein).

### Genotype–environment associations

GEAs were determined by five different methods, including GDM, LFMM, SFA, GLM where population membership (Q) served as a covariate, and last MLM where the average relationship was estimated by kinship (Q + k). Table 3 presents the significance levels at *p* ≤ 0.05, *p* ≤ 0.01 and *p* ≤ 0.001 for all SNPs for each analysis.

**Table 3:**
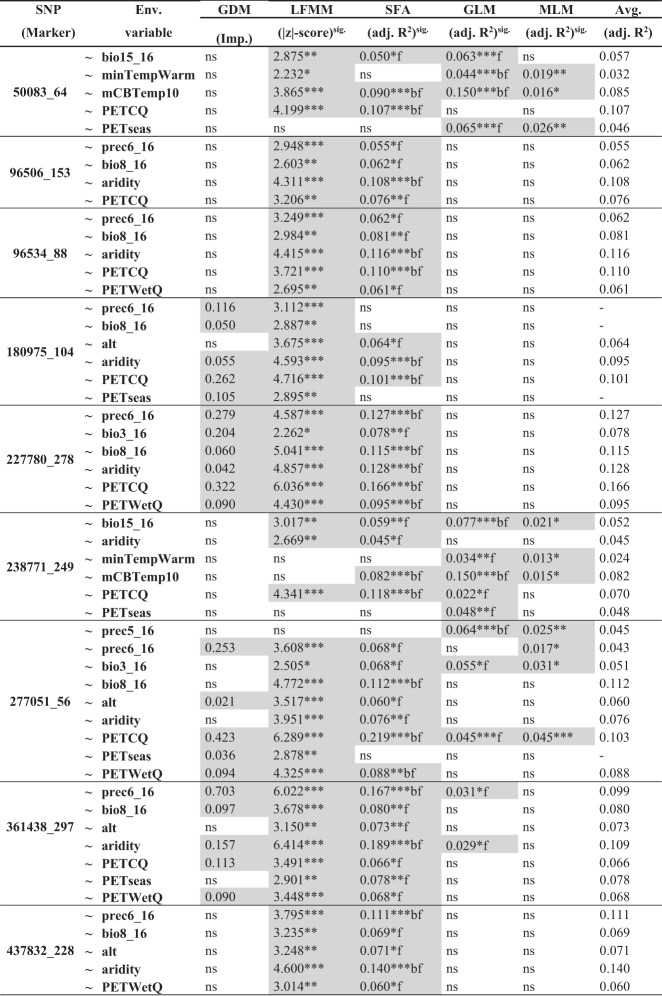
Outlier SNP markers associated with environmental variables using GDM, LFMM, SFA, GLM and MLM regression approaches.

SNPs that are associated with at least two different methods are marked in dark grey.

*p ≤ 0.05.

**p ≤ 0.01.

***p ≤ 0.001.

b: Bonferroni adjusted, *p* ≤ 0.05.

f: FDR adjusted, *p* ≤ 0.05.

*ns* non-significant.

By using the nine robust SNPs, a total of 102 significant marker-environment associations were detected, 21 with GDM, 48 with the LFMM, 9 with SFA and 14–10 with the GLM and MLM. However, similar to outlier analysis, we considered only those marker-environment associations that were detected with at least two different methods. Among the 53 jointly detected associations, the significance levels were different and ranged from 0.05 to 0.001.

**Fig. 4:**
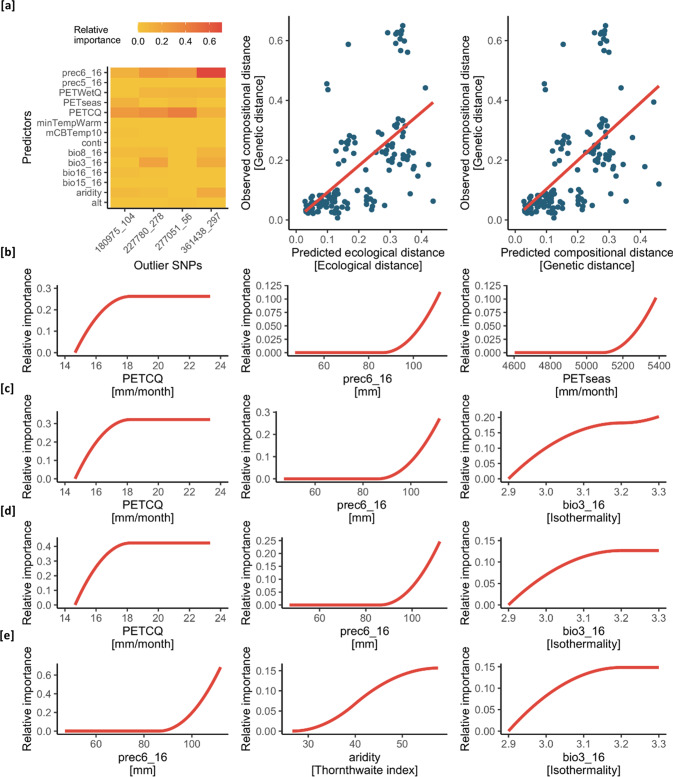
Associated outlier SNPs revealed by the matrix regression approach of GDM (Fitzpatrick et al. 2021). **a** Heatmap of the relative importance of each environmental predictor (darker colours indicate higher importance) and the relationship of ecological and genetic distances (left: relationship between predicted ecological distance and observed compositional dissimilarity; right: predicted versus observed genetic distance). **b–e** The three most important environmental predictors for each of the four outlier SNPs (180975_104, 227780_278, 277051_56, 361438_297), where associations were detected. Variable abbreviations are as explained in Table S6.

The GDM method found relationships only for four SNPs (180975_104, 227780_278, 277051_56, 361438_297). In these relationships, PETCQ, prec6_16, bio3_16 were the most frequent, and presented the highest relative importance (‘highest magnitude of biological change’; Fitzpatrick and Keller 2015) (Fig. 4). The rate of change in allele frequencies (shape of function) were different in each environmental variable. For all SNPs, in decreasing importance, PETCQ showed rapid change in allele frequencies at the beginning of the spline, while no change elsewhere. On the other hand, prec_6_16 showed rapid turnover at the end of the gradient. In case of bio3_16, the frequency of change was gradually increasing (Fig. 4).

The LFMM revealed associations for all nine outlier SNPs, in these |z | -score values ranged from 2.232 to 6.414 (S.D.: 4.182). The highest |z | -score values (i.e. the highest significance of an environmental effect) were found for three SNPs, namely for 227780_278, 277051_56 and 361438_297, in association with PETCQ (6.036 and 6.289), prec6_16 (6.022) and aridity (6.414). The single-factor analysis (SFA) was similar to LFMM, where the LFMM |z | -scores values were high, the SFA adjusted *R*^2^ values were also high. Thus, the highest *R*^2^ values were detected for 227780_278, 277051_56 and 361438_297, in association with PETCQ (0.166 and 0.219 *R*^2^), prec6_16 (0.167 *R*^2^) and aridity (0.189 *R*^2^), and explaining 16.6–21.9% of variance (Table 3).

The GLM and MLM detected much less numbers of associations (14 and 10), also the significance thresholds were largely different. The GLM detected associations with significant FDR and Bonferroni-adjusted thresholds (*p* values ranging from 0.05 to 0.001), however, the same associations detected by MLM, were not significant with FDR and Bonferroni-adjusted significance thresholds. The highest *R*^2^ values were detected for 50083_64 and 238771_249 in association with mCBTemp10 (both 0.150 *R*^2^). MLM, similarly to LFMM and SFA, revealed the highest significant association between 277051_56 and PETCQ (0.045 *R*^2^).

Altogether, the environmental variables explained 2.4–16.6% of the genetic variation (Table 3; average adjusted *R*^2^). SNP 50083_64, 180975_104 and 227780_278 was explained chiefly by PETCQ (avg. *R*^2^_adj_ = 10.7, 10.1 and 16.6%), 96506_153, 96534_88, 361438_297 and 437832_228 by aridity (avg. *R*^2^_adj_ = 10.8, 11.6, 10.9 and 14.4%), 238771_249 by mCBTemp10 (avg. *R*^2^_adj_ = 8.2%), 277051_56 by bio8_18 (avg. *R*^2^_adj_ = 11.2%). By summarizing the environmental predictors, in decreasing order, aridity (81.7%), PETCQ (79.9%), bio8_16 (51.9%) and prec6_16 (49.7%) explained the highest amount of the genetic variation, thus considered to be the most important (Tables 3, S7).

## Discussion

Previous studies on *Q. petraea* suggest on the one side generally high genetic diversity in the Balkan region due to the large number of refugia in this area and the high environmental heterogeneity (Zanetto and Kremer 1995; Gömöry et al. 2001; Tzedakis 2004), on the other side differential population responses to climate change (Sáenz-Romero et al. 2017). Although populations possess characteristics that can facilitate adaptation (prolific seed production and masting, high levels of genetic diversity, extensive gene flow) (Kremer 2016), they are becoming detached from the local climatic conditions to which they have previously adapted (Cheaib et al. 2012).The results of our study may provide information on how, despite continuous gene flow, populations from this area continued to differentiate as they successfully adapted to their new distinct environments. This may also suggest that adaptation to the climate might have occurred via many changes in the frequency of alleles for genes related to moisture deficit, temperature, and precipitation.

One important step of our method consisted of mapping our short reads to the *Q. robur* reference genome. Restriction-site-associated DNA sequencing or RAD-seq is increasingly used to identify large numbers of single-nucleotide polymorphisms (SNPs) (Allendorf et al. 2010; Davey and Blaxter 2010). Certainly, for the identification of genes under selection, the functional annotation of these genes and their location in the species’ genome are possible only through sequencing of the reference genome of a given, or a closely related species. Lacking a *Q. petraea* genome, we mapped our paired-end reads to the haploid version of the *Q. robur* genome (PM1N; http://www.oakgenome.fr; Plomion et al. 2018). After filtering, the short-read sequences were mapped onto the genomic reference with an average success rate of 92.43%. This percentage was relatively high considering that López de Heredia et al. (2020) managed to align their *Quercus suber* L. filtered reads to only 67.8% of the available *Q. suber* genome assembly. However, since PstI/MspI were selected as restriction enzymes in both studies, the mapping difference can be attributed to the different types of sequencing [single-end in the work of López de Heredia et al., paired-end in the work of Tóth et al. (2021)]. Another reason for the difference may be attributed to the different lengths and quality scores of the reads [mean quality score of at least 30 and length of at least 200 bp in our previous work, 20 and at least 20 bp in the work of López de Heredia et al. (2020)]. Finally, we must not forget the fact that the reference genomes assemblies were also different. Mapping our reads to the *Q. robur* genome allowed precise location of many of the loci in specific genes of known function.

### Population structure and diversity

Three main clusters were identified based on our results. The geographic extent of all clusters indicated a north–southeast gradient. The first cluster (Cluster 1) (yellow cluster in Fig. 2a) is dominant in continental regions, while the second cluster (Cluster 2) (blue) was found mainly in Central Europe (Fig. 2d). The southernmost Mediterranean group proved to be the third cluster (Cluster 3) (red) positioned at the border of Black Sea region. This geographical distribution corresponds with results from the work of both Zanetto and Kremer (1995) and Petit et al. (1993), in which longitudinal appeared to be more pronounced than latitudinal gradients. Regarding current knowledge of potential glacial refugia, in case of oaks, the western Balkans constituted a major refugium during the glacial periods (Bennett et al. 1991; Bordács et al. 2002; Petit et al. 2002). Recolonisation pathways probably followed a north-western postglacial migration route from the Balkans and Black Sea refugia. (Zanetto and Kremer 1995). These recolonization patterns may explain not only the north–southeast gradient but also the tendency to increase in probability towards the west of Cluster 3. The highly admixed nature of Cluster 2 (populations of HU and BH) could be a consequence of a transition zone, shown not only by Hewitt (1993) but also by Zanetto and Kremer (1995). The fact that Cluster 3 not only showed the highest differentiation but was also positioned on a separate branch with a much greater genetic distance from Clusters 1 and 2 also supports this assumption. We observed moderate levels of genetic diversity and allelic richness, with balanced values at the population level and no private alleles. As distribution of genetic diversity within and among populations is a function of the rate of gene flow (Bruschi et al. 2003), in our opinion this result can be attributed to different processes. First, woody species with large geographic ranges, outcrossing breeding systems, and wind-facilitated seed dispersal show less variation among populations than woody species with other combinations of traits (Hamrick et al. 1992; Hamrick and Godt 1996). Another possible interpretation of the observed differentiation pattern involves consequences of rapid migration. Oak species that expanded rapidly over large distances usually exhibit widely distributed haplotypes (Pham et al. 2017; Sork et al. 2016). Introgression of genes from *Q. pubescens* may be another reason the lack of differentiation (Bruschi et al. 2003). Rare alleles might have been lost because of selection pressures or during population bottlenecks (Allendorf 1986). Being associated with decreased fitness, selection against rare alleles occurred where viability selection was strongest at optimal growing sites (Bush and Smouse 1992). Of all populations, BU4 showed the lowest diversity values. Our sampling in Bulgaria was designed to cover the western Balkan, Rila, Rhodope and Strandzha Mts., geographic regions corresponding to the major genetic clusters. The BU4 population originates from the Strandzha Mts., as can be traced from the EUFORGEN distribution map (http://www.euforgen.org/species/quercus-petraea/), and this population is located at the eastern edge of the Black Sea refugium and in the eastern part of the current European distribution range of the species. Additionally, individuals of this region are considered to be a distinct subspecies in the Euro+Med Plantbase (http://ww2.bgbm.org/EuroPlusMed/query.asp) and on The Plant List (http://www.theplantlist.org), *Q. petraea* subsp. *iberica* (Steven ex M. Bieb.) Krassiln., and Schwarz (1993) considered it a separate species: *Q. polycarpa* Schur. (Schwarz 1993). The low level of intrapopulation diversity of the BU4 population may be explained by genetic drift (Newman and Pilson 1997), anthropogenic activities or by the successive bottlenecks that occurred at the head of the migration front (Dumolin-Lapegue et al. 1997), which may have reduced diversity during the eastward expansion from this refugium. It should also be noted that the lower genetic diversity observed could be the consequence of the stringent filtering strategy that is commonly applied for SNP filtering and quality control in GEA studies (DeSilva and Dodd 2020).

### Biological functions of outliers under selection

Local adaptation in natural populations may arise from differential selection pressures across heterogeneous environments (De La Torre et al. 2019). Therefore, different combinations of alleles might be favoured in these different environments and maintained as stable polymorphisms or experience partial sweeps due to selection acting on already standing variation (Hermisson and Pennings 2005). Our sampled populations experience different environmental conditions with different adaptations and selection pressures specific to their local habitats. Thus, we presumed that these populations evolve traits that provide an advantage in their local environment, and we performed different outlier detection methods, as these analyses can screen numerous markers in the genome to identify candidate genes for further investigation (Narum and Hess 2011). During our survey, as robust outliers, we detected nine SNPs located at different loci at six different chromosomes, and the *F*_ST_ values computed suggested positive (diversifying) selection. Those SNPs that were presented in NCBI databases resided in four different functional regions. Two SNPs found in a gene encoding the uncharacterized protein 115976593 (NCBI ID), one in bHLH162 transcription factor, one in hydroxymethylglutaryl-CoA synthase coding region, and one in a hypothetical protein coding region (possible double-strand damage repair). No biological function can be associated to uncharacterized proteins, however, bHLH transcription factor is related to NPF genes (Zhao et al. 2021), which have functions in stomatal opening and contributes to drought susceptibility in *Arabidopsis sp*. (Guo et al. 2003). Hydroxymethylglutaryl-CoA synthase has role in the biosynthesis of secondary metabolites under drought stress (Haider et al. 2017), by regulating the synthesis of mevalonic acid, the precursor of isoprenoid compounds (Bach 1986), that plays a role in various physiological processes, including cell membrane fluidity, plant defence under stress caused by environmental factors such as drought or high temperatures (Dani et al. 2014; Tattini et al. 2015).

### Precipitation as the strongest possible determinant of adaptation in *Quercus petraea*

Drought stress and temperature variation impose limitations on the survival, growth, and productivity of many forest tree species, and the survival of sessile oak is also affected by these two climatic factors. Evidenced by high level of genetic differentiation observed in common garden experiments, oak populations have responded to climatic selection during historical global warming after the last glaciation (Kremer et al. 2014; Torres-Ruiz et al. 2019). Previous studies have also suggested differential responses to temperature and moisture across geographically distant populations of the species (Bruschi et al. 2003; Müller and Gailing 2019; Mátyás 2021), with higher population differentiation, for example, compared to that of *Quercus robur* L. (Kremer and Petit 1993).

In our study, nine SNPs (0.34%) showed signatures of selection according to differentiation outlier analyses. The observed proportion was lower than obtained in other studies on temperate broadleaf forest trees (Derory et al. 2010; Alberto et al. 2013; Csilléry et al. 2014; De Kort et al. 2014; Sork et al. 2016; Temunović et al. 2020).

Caution must be exercised when interpreting our results, taking into account the potential for false positive selection signatures (Excoffier et al. 2009; De Villemereuil et al. 2014; Whitlock and Lotterhos 2015; Hoban et al. 2016) and past introgression events (Bierne et al. 2011; Guichoux et al. 2013). Should be also noted that the proportion we obtained from SNPs detected by two different approaches, including our restriction site-associated DNA sequencing method, was based on only a few SNPs per gene region (Luikart et al. 2003). Our results revealed 53 associations between markers and environment, with significance levels ranging from *p* ≤ 0.05 to 0.001. The variations seen in the analyses are due to differences in the methods used to account for demographic history or neutral genetic structure, which results in different statistical strengths for different sampling schemes or complicated demographic situations (De Mita et al. 2013; Forester et al. 2018; Aguirre-Liguori 2021). Altogether, the environmental variables explained 2.4–16.6% of the genetic variation. SNP 50083_64 (hypothetical protein), 180975_104 (transcription factor bHLH162), 227780_278 (F-box protein At-B) and 437832_228 (hydroxymethylglutaryl-CoA synthase) associated with aridity, 238771_249 (uncharacterized protein) with mCBTemp10. By summarizing the environmental predictors, in decreasing order, aridity (81.7%), PETCQ (79.9%), bio8_16 (51.9%) and prec6_16 (49.7%) explained the highest amount of the genetic variation. In general, our analyses discovered stronger connections with environmental changes than prior studies. Although it is challenging to compare with our study owing to the use of distinct markers, analytical techniques, and climate variables utilized, Homolka et al. (2013) found significant population differentiation for three genes with strong correlations with the local temperature–precipitation regime, Neophytou et al. (2015) did not detect any significant relationships between the considered environmental variables and neutral genetic variation, only Rellstab et al. (2016) found 521 associations in 224 SNPs. The different signal power we noticed may be somewhat attributable to methodological factors, given that our analysis employed a different dataset and statistical methods than Homolka et al. (2013) and Neophytou et al. (2015). Additionally, our selection of SNP loci was more narrowly focused on climate factors than Rellstab et al. (2016), since we aimed for genes likely implicated in traits that are susceptible to climate shifts, especially drought-related ones. Our result also provide evidence for adaptation to drought detected in the provenance study of Bert et al. (2020), who observed provenances originating from sites with wet summers displaying the strongest responses to summer drought, particularly in the driest common garden.

The above list of significant marker-environment associations clearly shows, that most variables are linked to aridity. To validate functional mechanisms underlying selection signatures, it is generally recommended to use annotations, which can provide additional support beyond relying solely on *F*_ST_ outlier and environmental association analyses, which are only correlative measures (Ahrens et al. 2018). As we previously mentioned when annotating the genes with significant environmental associations, according to literature bHLH transcription factor (marker 180975_104) is related to functions in stomatal opening and contributes to drought susceptibility (Babitha et al. 2015; Qian et al. 2021), marker 227780_278 resides in a region encoding F-box protein At-B, whose transcription is induced by abscisic acid, which regulates the regulation of abiotic stress responses (Sah et al. 2016; Park and Kim 2021) and marker 437832_228 is in a region encoding hydroxymethylglutaryl-CoA synthase with role in the biosynthesis of secondary metabolites under drought stress (Ghasemi et al. 2019; Rogowska and Szakiel 2020). By matching gene functions with relevant climatic factors we can provide biological significance to the genetic basis of local adaptation and the impact of climate on divergent selection among our studied sessile oak populations. Nonetheless, assigning functions to specific candidates in nonmodel species should be approached with caution, and further association genetics and functional studies are needed to validate their role in adaptive traits, as recommended by Pavlidis et al. (2012).

## Conclusion

While sessile oak populations are well studied in Europe, in the Central-Eastern European region, including the Balkan Peninsula it is seldom tested with genome-wide genetic markers. The identification of genetic variants within populations of this region may hold the key to facilitating the sessile oak’s adaptation to the deleterious effects of modern climate change, thus underscoring the crucial significance of further investigation in this field. Through our examination, we found molecular evidence indicating that important climate-related factors, such as drought, may have influenced the adaptive divergence of sessile oak populations in the Central-Eastern European region. Moreover, our results suggest that these populations may contain beneficial genetic variants that could aid the species in responding to the rapidly changing climate. In light of these findings, it is vital that European forest tree species conservation and management programmes incorporate the Central-Eastern European sessile oak populations, including facilitated gene flow, into their strategies to combat the significant threat that climate change poses to forest tree populations.

## Supplementary information

Supplementary Material

## Data Availability

The genomic dataset analysed during the current study is available in Tóth et al. (2021). The dataset can also be accessed at 10.5281/zenodo.3908963. The sequences of each outlier loci are available at 10.5281/zenodo.7763329.
