# Supplementary material for: Key triggers of adaptive genetic variability of sessile oak [Q. petraea (Matt.) Liebl.] from the Balkan refugia: outlier detection and association of SNP loci from ddRAD-seq data
Source: Heredity (Edinb). 2023 Jun 14;131(2):130–44. doi: 10.1038/s41437-023-00629-2 (PMC10382515; doi:10.1038/s41437-023-00629-2)
Supplement: Supplementary file 1 — Supplementary Material [file 41437_2023_629_MOESM1_ESM.docx]

**Key triggers of adaptive genetic variability of sessile oak [*Q. petraea* (Matt.) Liebl.] from the Balkan refugia: outlier detection and association of SNP loci from ddRAD-seq data**

**Supplementary information**

**Genotyping**

Briefly, genomic DNA was extracted from leaves (Dumolin et al. 1995) and quantified by using the Qubit dsDNA BR Assay Kit and a Qubit 3.0 Fluorometer (Thermo Fisher Scientific, Waltham, MA, USA). DNA samples were double digested with *PstI* and *MspI* (FastDigest restriction enzymes; Thermo Fisher Scientific, Waltham, MA, USA) based on the study of Cumer et al. (2018). Fragments were double-sided size selected using KAPA PureBeads (Roche, Basel, Switzerland) to isolate fragments in the range of 300–600 bp. After quantification, inserts (3 ng) were ligated to adapters using T4 DNA Ligase according to the manufacturers’ protocol (Thermo Fisher Scientific, Waltham, MA, USA). Products were purified using 0.8 vol KAPA PureBeads (Roche, Basel, Switzerland) and amplified by PCR with NEBNext Multiplex Oligos for Illumina (Dual Index Set 1; New England Biolabs, Ipswich, MA, USA) and KAPA HiFi Hotstart Ready Mix (Roche, Basel, Switzerland). The quality and quantity of the amplicon library were determined by using a High-Sensitivity DNA1000 ScreenTape system with 2200 Tapestation (Agilent Technologies, Santa Clara, CA, USA) and a dsDNA HS Assay Kit with a Qubit 3.0 Fluorometer (Thermo Fisher Scientific, Waltham, MA, USA), respectively. Pooled libraries were diluted to 10 pM for 2x300 bp paired-end sequencing on the MiSeq Sequencing System platform.

**Tables and Figures**

**Tables**

**Table S1**: Details of the 18 natural populations of *Quercus petraea* in the Central-Eastern European region, including the Balkan Peninsula: ID-code; country; geographic region, mean sampling altitude at each site (m a.s.l.); number of samples; latitude and longitude (in decimal degrees)

| **Pop. ID** | **Country** | **Region** | **Alt. (m a.s.l)** | **n** | **Lat.** | **Long.** |
| --- | --- | --- | --- | --- | --- | --- |
| AL1 | Albania | Djeravica Mt. | 454 | 10 | 42.407 | 20.168 |
| RO1 | Romania | Gurghiu Mts. | 438 | 10 | 46.694 | 24.692 |
| RO2 | Romania | Fagaras Mts. | 508 | 10 | 45.839 | 24.007 |
| RO3 | Romania | Fagaras Mts. | 455 | 10 | 44.853 | 24.793 |
| SE1 | Serbia | Fruska Gora Mts. | 208 | 10 | 45.172 | 19.839 |
| SE2 | Serbia | Rudnik Mts. | 578 | 10 | 44.172 | 20.488 |
| SE3 | Serbia | Stovoli Mts. | 414 | 10 | 43.667 | 20.603 |
| KO1 | Kosovo | Blinaja | 787 | 10 | 42.513 | 20.968 |
| BU1 | Bulgaria | Balkan Mts. | 601 | 10 | 42.98 | 23.826 |
| BU2 | Bulgaria | Rila Mts. | 950 | 9 | 42.353 | 23.686 |
| BU3 | Bulgaria | Rhodope Mts. | 1172 | 10 | 42.073 | 23.977 |
| BU4 | Bulgaria | Strandzha Mts. | 211 | 10 | 42.117 | 27.766 |
| HU1 | Hungary | Kőszeg Mts. | 351 | 10 | 47.371 | 16.524 |
| HU2 | Hungary | Bakony Mts. | 374 | 10 | 46.835 | 17.319 |
| HU3 | Hungary | Mecsek Mts. | 372 | 10 | 46.200 | 18.432 |
| BH1 | Bosnia and Herzegovina | Kozara Mts. | 311 | 10 | 45.157 | 16.979 |
| BH2 | Bosnia and Herzegovina | Javorova Mt. | 440 | 10 | 44.662 | 17.706 |
| BH3 | Bosnia and Herzegovina | Maglic Mt. | 985 | 10 | 43.476 | 18.946 |

**Table S2**: Mean number and percentage (%) of successfully mapped reads for each individual resulting from reference genome mapping. Population abbreviations are as explained in Tóth et al. 2021.

| **Population** | **Individual ID** | **Mapped reads** | **Percentage** |
| --- | --- | --- | --- |
| AL1 | AL1-1 | 327454 | 95.30 |
| AL1 | AL1-10 | 330489 | 95.76 |
| AL1 | AL1-2 | 362662 | 95.06 |
| AL1 | AL1-3 | 360714 | 94.17 |
| AL1 | AL1-4 | 303645 | 91.74 |
| AL1 | AL1-5 | 318762 | 94.15 |
| AL1 | AL1-6 | 349797 | 95.80 |
| AL1 | AL1-7 | 342247 | 94.51 |
| AL1 | AL1-8 | 314385 | 95.08 |
| AL1 | AL1-9 | 351940 | 95.71 |
| BU1 | BU1-1 | 360882 | 93.91 |
| BU1 | BU1-10 | 342508 | 94.27 |
| BU1 | BU1-2 | 359587 | 93.00 |
| BU1 | BU1-3 | 350159 | 95.11 |
| BU1 | BU1-4 | 305521 | 94.59 |
| BU1 | BU1-5 | 387826 | 94.73 |
| BU1 | BU1-6 | 361732 | 90.63 |
| BU1 | BU1-7 | 373849 | 95.66 |
| BU1 | BU1-8 | 313173 | 93.50 |
| BU1 | BU1-9 | 340069 | 94.15 |
| KO1 | KO1-1 | 362701 | 93.60 |
| KO1 | KO1-10 | 341875 | 95.09 |
| KO1 | KO1-2 | 334159 | 95.30 |
| KO1 | KO1-3 | 353562 | 91.56 |
| KO1 | KO1-4 | 429811 | 94.33 |
| KO1 | KO1-5 | 385202 | 93.85 |
| KO1 | KO1-6 | 319762 | 87.25 |
| KO1 | KO1-7 | 337045 | 89.46 |
| KO1 | KO1-8 | 363282 | 94.97 |
| KO1 | KO1-9 | 360823 | 94.14 |
| BH1 | QPET-BH1-1 | 402846 | 95.92 |
| BH1 | QPET-BH1-10 | 576537 | 95.68 |
| BH1 | QPET-BH1-2 | 620735 | 93.93 |
| BH1 | QPET-BH1-3 | 320573 | 96.32 |
| BH1 | QPET-BH1-4 | 429931 | 96.59 |
| BH1 | QPET-BH1-5 | 352796 | 96.12 |
| BH1 | QPET-BH1-6 | 445693 | 89.57 |
| BH1 | QPET-BH1-7 | 406594 | 96.18 |
| BH1 | QPET-BH1-8 | 376399 | 96.28 |
| BH1 | QPET-BH1-9 | 451282 | 96.13 |
| BH2 | QPET-BH2-1 | 319498 | 96.47 |
| BH2 | QPET-BH2-10 | 341874 | 93.57 |
| BH2 | QPET-BH2-2 | 368764 | 95.05 |
| BH2 | QPET-BH2-3 | 409732 | 94.04 |
| BH2 | QPET-BH2-4 | 409925 | 91.44 |
| BH2 | QPET-BH2-5 | 381544 | 91.41 |
| BH2 | QPET-BH2-6 | 407581 | 93.00 |
| BH2 | QPET-BH2-7 | 326414 | 92.13 |
| BH2 | QPET-BH2-8 | 636899 | 95.01 |
| BH2 | QPET-BH2-9 | 215607 | 92.89 |
| BH3 | QPET-BH3-1 | 277987 | 87.23 |
| BH3 | QPET-BH3-10 | 330840 | 90.79 |
| BH3 | QPET-BH3-2 | 337320 | 85.28 |
| BH3 | QPET-BH3-3 | 383439 | 91.69 |
| BH3 | QPET-BH3-4 | 354592 | 90.42 |
| BH3 | QPET-BH3-5 | 429673 | 93.37 |
| BH3 | QPET-BH3-6 | 455845 | 87.69 |
| BH3 | QPET-BH3-7 | 222650 | 88.11 |
| BH3 | QPET-BH3-8 | 296124 | 86.14 |
| BH3 | QPET-BH3-9 | 248076 | 91.64 |
| BU2 | QPET-BU2-1 | 332712 | 92.80 |
| BU2 | QPET-BU2-10 | 174 | 80.93 |
| BU2 | QPET-BU2-2 | 343927 | 94.21 |
| BU2 | QPET-BU2-3 | 368802 | 94.36 |
| BU2 | QPET-BU2-4 | 324992 | 93.52 |
| BU2 | QPET-BU2-5 | 377560 | 89.04 |
| BU2 | QPET-BU2-6 | 377805 | 92.77 |
| BU2 | QPET-BU2-7 | 297095 | 92.89 |
| BU2 | QPET-BU2-8 | 445079 | 90.72 |
| BU2 | QPET-BU2-9 | 212730 | 90.33 |
| BU3 | QPET-BU3-1 | 354429 | 95.83 |
| BU3 | QPET-BU3-10 | 443505 | 93.09 |
| BU3 | QPET-BU3-2 | 404668 | 95.75 |
| BU3 | QPET-BU3-3 | 421255 | 96.27 |
| BU3 | QPET-BU3-4 | 367044 | 87.21 |
| BU3 | QPET-BU3-5 | 300315 | 95.21 |
| BU3 | QPET-BU3-6 | 555664 | 93.85 |
| BU3 | QPET-BU3-7 | 228885 | 94.68 |
| BU3 | QPET-BU3-8 | 330339 | 94.94 |
| BU3 | QPET-BU3-9 | 405263 | 96.04 |
| BU4 | QPET-BU4-1 | 394406 | 88.14 |
| BU4 | QPET-BU4-10 | 381411 | 94.63 |
| BU4 | QPET-BU4-2 | 364564 | 90.51 |
| BU4 | QPET-BU4-3 | 307323 | 91.04 |
| BU4 | QPET-BU4-4 | 483927 | 92.94 |
| BU4 | QPET-BU4-5 | 298664 | 93.98 |
| BU4 | QPET-BU4-6 | 312965 | 92.70 |
| BU4 | QPET-BU4-7 | 365593 | 93.26 |
| BU4 | QPET-BU4-8 | 417030 | 94.59 |
| BU4 | QPET-BU4-9 | 356549 | 95.24 |
| HU1 | QPET-HU1-1 | 335759 | 96.48 |
| HU1 | QPET-HU1-10 | 338226 | 96.27 |
| HU1 | QPET-HU1-2 | 350812 | 95.99 |
| HU1 | QPET-HU1-3 | 338108 | 93.17 |
| HU1 | QPET-HU1-4 | 316629 | 95.44 |
| HU1 | QPET-HU1-5 | 239735 | 94.28 |
| HU1 | QPET-HU1-6 | 305015 | 96.15 |
| HU1 | QPET-HU1-7 | 314002 | 95.33 |
| HU1 | QPET-HU1-8 | 347449 | 96.39 |
| HU1 | QPET-HU1-9 | 345659 | 96.15 |
| HU2 | QPET-HU2-1 | 318549 | 90.34 |
| HU2 | QPET-HU2-10 | 432732 | 91.74 |
| HU2 | QPET-HU2-2 | 324244 | 90.75 |
| HU2 | QPET-HU2-3 | 230883 | 86.50 |
| HU2 | QPET-HU2-4 | 298682 | 88.36 |
| HU2 | QPET-HU2-5 | 302401 | 87.21 |
| HU2 | QPET-HU2-6 | 322155 | 87.51 |
| HU2 | QPET-HU2-7 | 285685 | 82.22 |
| HU2 | QPET-HU2-8 | 306714 | 89.22 |
| HU2 | QPET-HU2-9 | 354869 | 88.93 |
| HU3 | QPET-HU3-1 | 279081 | 96.27 |
| HU3 | QPET-HU3-10 | 315802 | 96.29 |
| HU3 | QPET-HU3-2 | 301176 | 96.30 |
| HU3 | QPET-HU3-3 | 331818 | 95.65 |
| HU3 | QPET-HU3-4 | 386843 | 96.33 |
| HU3 | QPET-HU3-5 | 368596 | 95.88 |
| HU3 | QPET-HU3-6 | 329606 | 95.94 |
| HU3 | QPET-HU3-7 | 370625 | 96.11 |
| HU3 | QPET-HU3-8 | 358262 | 95.44 |
| HU3 | QPET-HU3-9 | 257318 | 96.63 |
| RO1 | RO1-1 | 293531 | 87.47 |
| RO1 | RO1-10 | 404919 | 83.86 |
| RO1 | RO1-2 | 316402 | 90.07 |
| RO1 | RO1-3 | 343875 | 86.02 |
| RO1 | RO1-4 | 366187 | 89.02 |
| RO1 | RO1-5 | 337991 | 85.26 |
| RO1 | RO1-6 | 344329 | 89.20 |
| RO1 | RO1-7 | 357536 | 89.17 |
| RO1 | RO1-8 | 340401 | 89.91 |
| RO1 | RO1-9 | 324391 | 85.17 |
| RO1 | RO2-1 | 352886 | 94.04 |
| RO1 | RO2-10 | 323926 | 94.94 |
| RO2 | RO2-2 | 321543 | 91.82 |
| RO2 | RO2-3 | 436774 | 94.52 |
| RO2 | RO2-4 | 403447 | 91.61 |
| RO2 | RO2-5 | 400180 | 93.55 |
| RO2 | RO2-6 | 356972 | 93.71 |
| RO2 | RO2-7 | 320241 | 93.59 |
| RO2 | RO2-8 | 349091 | 88.22 |
| RO2 | RO2-9 | 404749 | 94.72 |
| RO3 | RO3-1 | 323009 | 88.96 |
| RO3 | RO3-10 | 430066 | 91.96 |
| RO3 | RO3-2 | 377799 | 84.44 |
| RO3 | RO3-3 | 357656 | 93.06 |
| RO3 | RO3-4 | 309419 | 88.17 |
| RO3 | RO3-5 | 401299 | 91.82 |
| RO3 | RO3-6 | 424206 | 86.59 |
| RO3 | RO3-7 | 421978 | 92.03 |
| RO3 | RO3-8 | 367104 | 89.68 |
| RO3 | RO3-9 | 374568 | 91.53 |
| SE1 | SE1-1 | 444213 | 91.42 |
| SE1 | SE1-10 | 288087 | 69.26 |
| SE1 | SE1-2 | 315566 | 74.17 |
| SE1 | SE1-3 | 388358 | 93.25 |
| SE1 | SE1-4 | 442663 | 91.49 |
| SE1 | SE1-5 | 411698 | 91.85 |
| SE1 | SE1-6 | 327012 | 85.62 |
| SE1 | SE1-7 | 405745 | 92.87 |
| SE1 | SE1-8 | 387390 | 87.24 |
| SE1 | SE1-9 | 432743 | 93.04 |
| SE2 | SE2-1 | 399355 | 95.74 |
| SE2 | SE2-10 | 412151 | 94.38 |
| SE2 | SE2-2 | 390619 | 91.99 |
| SE2 | SE2-3 | 408637 | 96.11 |
| SE2 | SE2-4 | 351117 | 95.36 |
| SE2 | SE2-5 | 393784 | 91.58 |
| SE2 | SE2-6 | 399838 | 95.59 |
| SE2 | SE2-7 | 383665 | 91.91 |
| SE2 | SE2-8 | 352131 | 94.77 |
| SE2 | SE2-9 | 370849 | 95.31 |
| SE3 | SE3-1 | 370106 | 95.05 |
| SE3 | SE3-10 | 333638 | 94.69 |
| SE3 | SE3-2 | 339464 | 94.65 |
| SE3 | SE3-3 | 341514 | 94.31 |
| SE3 | SE3-4 | 413844 | 94.94 |
| SE3 | SE3-5 | 388056 | 95.11 |
| SE3 | SE3-6 | 358264 | 94.67 |
| SE3 | SE3-7 | 309402 | 95.72 |
| SE3 | SE3-8 | 344784 | 94.10 |
| SE3 | SE3-9 | 321662 | 89.75 |

**Table S3**: Mean percentage (%) of successfully mapped, paired and singleton reads by population resulting from reference genome mapping. Population abbreviations are as explained in Tóth et al. 2021.

| **Population** | **Mapped reads** | **Paired reads** | **Singleton reads** |
| --- | --- | --- | --- |
| AL1 | 94.73 | 88.43 | 0.88 |
| BU1 | 93.96 | 87.77 | 0.90 |
| KO1 | 92.96 | 86.65 | 0.94 |
| BH1 | 95.27 | 88.64 | 0.86 |
| BH2 | 93.50 | 85.74 | 0.98 |
| BH3 | 89.24 | 81.85 | 1.11 |
| BU2 | 91.16 | 83.46 | 1.76 |
| BU3 | 94.29 | 87.81 | 0.79 |
| BU4 | 92.70 | 84.98 | 0.93 |
| HU1 | 95.57 | 87.12 | 1.20 |
| HU2 | 88.28 | 80.30 | 1.25 |
| HU3 | 96.08 | 87.84 | 1.80 |
| RO1 | 87.52 | 81.56 | 0.99 |
| RO2 | 93.07 | 86.87 | 0.91 |
| RO3 | 89.82 | 83.25 | 0.97 |
| SE1 | 87.02 | 80.57 | 1.15 |
| SE2 | 94.27 | 87.62 | 0.88 |
| SE3 | 94.30 | 87.83 | 0.87 |
| **mean** | **92.43** | **85.46** | **1.07** |

**Table S4**: Genetic diversity of the 18 natural populations of *Q. petraea* and the mean diversity estimated for each genetic cluster detected by fastStructure (Raj et al., 2014). Population abbreviations are as explained in Tóth et al. 2021.

| **Population** | **H_o_** | **H_e_** | **Ar** | **PA** | ***F*_IS_** |
| --- | --- | --- | --- | --- | --- |
| AL1 | 0.199 | 0.213 | 1.715 | 0 | 0.044 |
| RO1 | 0.210 | 0.216 | 1.726 | 0 | 0.023 |
| RO2 | 0.203 | 0.213 | 1.719 | 0 | 0.037 |
| RO3 | 0.202 | 0.216 | 1.716 | 0 | 0.051 |
| SE1 | 0.208 | 0.223 | 1.746 | 0 | 0.051 |
| SE2 | 0.202 | 0.216 | 1.721 | 0 | 0.050 |
| SE3 | 0.202 | 0.213 | 1.696 | 0 | 0.037 |
| KO1 | 0.205 | 0.217 | 1.722 | 0 | 0.042 |
| BU1 | 0.203 | 0.216 | 1.722 | 0 | 0.041 |
| BU2 | 0.192 | 0.205 | 1.673 | 0 | 0.048 |
| BU3 | 0.191 | 0.208 | 1.688 | 0 | 0.058 |
| BU4 | 0.166 | 0.181 | 1.570 | 0 | 0.062 |
| HU1 | 0.193 | 0.205 | 1.651 | 0 | 0.040 |
| HU2 | 0.204 | 0.219 | 1.719 | 0 | 0.051 |
| HU3 | 0.204 | 0.219 | 1.718 | 1 | 0.051 |
| BH1 | 0.202 | 0.215 | 1.704 | 0 | 0.040 |
| BH2 | 0.198 | 0.210 | 1.688 | 0 | 0.043 |
| BH3 | 0.199 | 0.212 | 1.698 | 0 | 0.050 |
| Cluster 1 | 0.201 | 0.214 | 1.712 | 0.000 | 0.044 |
| Cluster 2 | 0.200 | 0.213 | 1.696 | 0.200 | 0.045 |
| Cluster 3 | 0.166 | 0.181 | 1.570 | 0.000 | 0.062 |
| *F*_IS_: inbreeding coefficient  Ar: allelic richness  PA: private alleles  H_e_: expected heterozygosity  H_o_: observed heterozygosity | | | | | |

**Table S5**: BLASTn/BLASTx searches for the nine loci detected at the outlier SNP analyses (on the example of AL1-1 sample).

| **Sample** | **Chr** | **Locus name** | **Sequence** | **Per. Identity** | **E-value** | **Query Cover** | **Accession** | **Description** |
| --- | --- | --- | --- | --- | --- | --- | --- | --- |
| AL1-1 | 2 | 50083 | GCAGCATATATAATTAGATCTTCAGCTGAAGCTGCTGAAATGGCGACTGAATAAACTGGAGCTTAATCTCGATCTCAACAATATATAACTAGTGGTTTTGTACCTGCTACCTCTTATGCTGCCTTCCCAACCTTTGCCTCCGATGCTAGCTCTCGACTTGGAAAGGATGCCACTGTCTTTGCCATAGGTGGTTATGGATCCACTCGGTCAACTGAAACTCCTGCCCGTGCCTCTGCCATTGGCTTTGCTGCATGCTCCGTTACTGGCTCTCTAAATGAAATTAGCCGTGCTGCGGGGTCTACTCAATCTGGGTCTGTCCCTAGTGGTGCTTAAACAAGTGCTGCTGCGGGAACCCGGTCAACCGCTACAAGTCCTGAGTCAAAAAGGTAAGCTCGGTTTCAATACCCTTCCTGTGTGCTTTGTTCCCTGTACTCTTGCCTTTTCCTCTCCTGCAGGCGGTGAATCAAGAGGATATGGATCTCCAATGAATGGCTCTCGATCTGGCTTAGATGCTGATTCGAATGCTGCCAGTCAATTGCTGGTGTTACAACCTATGCTACCTTCTCTCCTGCTACCGGTGCTTATTTGACTGGGACTGGAAGGAATGCTACTGACTCTCGATCTCGATCTAAATCGCCAACTGGATAGGGATCATCTGCTCGCATCTCCGACAGGATTTGGAACCTTATCG | 92.41% | 2,00E-37 | 54% | POE44728.1 | hypothetical protein CFP56_76761 [Quercus suber] |
| AL1-1 | 2 | 96506 | GCAGGGAGATTCTGATCAGCAGTGATTTCATCAATGGTAACTGCTTCAGTGCCCAGGGATTTGGATTCCAACACTGTTCCAGAAGCTTCTTTGATTCCTTCTGTTCTGTCTGCATCCAAATTTTCCACTCTTAAACTTTGCCCAGTCTCATTCGTTTCTTCAACACCATGATATTTGGACTGGAAAACTGCATCAGAAACTGTTTTGGTTTTCTCATCTGCATCTGGTTTCTCCGGAGTTACAGTCTCTGTCTCTATATCATTGTGTTTCATATTTGGGAGTTGAGACACCAAGCTAAAGGAAGTTTTTTCAGGAGTCATGCTGCCAACTTCTTCAGCATGTGGACAAGGCTC | 99.15% | 9,00E-178 | 100% | XM_031097988.1 | Quercus lobata titin homolog (LOC115976593), transcript variant X36, mRNA |
| AL1-1 | 2 | 96534 | GCAGGGAGAATCTGAAAACCTGGCTCCAAGAACAGAAGCTGCAATAGAGAAAAGAGAAGAGGAATCTTACATCAAAGTAACTGAGGACAACAATGATAATGAACAAGCTGAAGTTACAGAAAACAGTGAAAAGGTAACACCAGAAGGTGGAGAAGATTCAGAGAAAATACTACAGGTAGATGAACCAGGACAGAGCATTGTAGGCATGGAAAATCGCAATGTTGACAGCCCTGAAGGTGAAGAAGATTCAGAACAAGCTTTACAGCGTGATGAACCACGAGAGACAAATCATGAGTTTGAAAGCCCCGGAGAAGTTCTTGATACTTCATTGGTGAACTCAGGGAAAGGTTTGCAGGGAGAAAGTACGAACCTGGCTCAAGCAGAAACTTCACAAGAGAAAAGAGATGAAGCCATTACTTCTGTCAAAGTTGCTGTGGACAATGATGACAATGACAAACTAGCTG | 99.14% | 0.0 | 100% | XM_031097988.1 | Quercus lobata titin homolog (LOC115976593), transcript variant X36, mRNA |
| AL1-1 | 5 | 180975 | GCAGCTCCATAACCCAATGCAGAATCCCCAACCTACAATTTGAAGCCAAAAATAGTCTTACATATTTTTCATTGAGCAAATGACAGTCTCTAAAAAGATTTAAACAAAGAGAATTGCCTAGCTACCTTAGAATGAATTGTGTGGAACACTGTATCTTCAACAACAGTGAAGCTGGCATTAACAATCTCAGCTCCCTCTTCATGAAGCACGCGAATAGTCTCATTGAACATAAACTGAAAATCTAACCCAGTTATTAAAACAACCTCTAAAGCAGAACCCACTTCGTGAATCTCAATTTGTGGTGGCTTAAAACCCATCGTCATTCCACAATCGGTGCTTGCGTTTGACTTTTCATGAATTCCCATTAGATTGTTTTTCTTCTCCTTCATTCTCTCCAACTTTGTCTGTAGCCTCTTTATATAGTTTGCAGCTTCATCTAGTTGATCCGGC | 100% | 3,00E-168 | 80% | XM_050431057.1 | Quercus robur transcription factor bHLH162 (LOC126725984), transcript variant X3, mRNA |
| AL1-1 | 6 | 227780 | GCAGTTCTAGATTACCTAGGAACTGTCATCGAGGTACTTCTACCTTGACCCCTGCTTCAATTATGTTCTTCCTTATTTATTTATGCTTCGTTTGTTTCGAAGTAAAATACTTTCAAATGTAAAATATTTTACATGTAAAAAATTTTACATGAAAATAATTTCAGTGAAGACATTTTCAAGTGTTTGGCATGACTAAGCACTTGAAAATGCAAGCACAAACCAACCACCACCAACAACCTCAAACCCAGCCACACAGCCGCCACCACCACCTACCATAAACTCAGCCATTACGGTCACAAATCCCAGCAACACCAAACCCAGCCAGCCACCACGACCTCCGCAACCATAACTACCTAGATTTGAGAGATGCGGCCTGAAAAAAATGAGGAAGAGGAGGAAGATCTGCACCGCATTACTGCTGCGACTTCCACACCGGAGACATCAAGGCTGACCGTCCTCACCACCAGCATAAGTCGGAGTCCCAAGTTACTGCCAGAAAGATGAGAAGAGAGAGGAGGGGGTTTAGGAGAAGAGGGA | (low coverage; 13%) | 4,00E-14 | 89.47% | XM_050394247.1 | Quercus robur uncharacterized LOC126697307 (LOC126697307), mRNA |
| AL1-1 | 6 | 238771 | GCAGCTCTTGCCGTTGAAGGAATAAGTGTTGCTTTGGCTTGATTGCTTTCACCAGGTCTAGCTAGGCTGGGAGTCGATTAGCCCGAGTTGTGTTAAGATAAGTGGCACTTGAATTTGAAGTAGCCATCGGCCAGGTCTTGGATGCTGAATTCTCGGTCTTACCACTTGTATCGATAGACCCACTTTCATTGAATGCTGAGGAGATTGATTGAAAAGAAAATCCTGATCCTGATCTTCGAACTTTCTCTCTGCTTGAAGTCAAAACAAAGCTTGAACCAGGTCACAGGGAAAGCTTTTCTTGCTAATCTGGTGATATCGTAGTAAAGGTAAAGTAGCCAAGCTAAGAGGAATCGATTTAGCAGTCCATACCAGGCTCCCGGTAGATTTAGGAGTGACCGGCAGTGAATGCCCG | 98.80% | 7,00E-120 | 60% | XM_050408112.1 | Quercus robur uncharacterized LOC126708314 (LOC126708314), mRNA |
| AL1-1 | 7 | 277051 | GCAGAAATTATTATCTTCCTATAGAACTTACATCAGACATCAACAACATTCTATTCATGCTATTTTACCTACAAGGGGTCGCAAGAAAAACAAAACCTGGGGCAAAACCATATCTCAAACATCCTATATACGAACATCTCAGTAAACACAGCTATTAAGATTAAAGGGGAAATTTTAGTAGAATGTACAAAATGCCTTTGAACCACAAACAGAAAAAAAANTGGTCTCATATCTCAAGCTTCCAAATGGTTACGAAGGTGCTGTATAGGAGGAAAGCTCAAACTTAGGCTGCTTTGTTAGAATTTACTGTTCTATTCATTCAAACTTTAACCATATAGATATATCATCCATTCCCACTGAAGTCAGACAGATAAAGGTGGGAATTCTTCTTCATCTTTCAAACGGAATGATTGTGCAATCCTACATGTCCAAAACAAAGAAACTTAAAAAATAGACCCATGTATGTCAGCTAGTACCG | 99.76% | 0.0 | 88% | XM_050388161.1 | Quercus robur uncharacterized LOC126692534 (LOC126692534), transcript variant X3, mRNA |
| AL1-1 | 9 | 361438 | GCAGCAAGGAAACAGAAAGGATCTACCGCAGTAGTCCTTCCCCCACCCAGTTAAGGGCCACATGAATCCTATAGCTTCTTTGGACAACTATTGCCGTCTTGTCTTCAAGTGCGATACAAGGAGATGACCACGTACTGTGATTATGCTTCCTGTAAGAACATCCCTTTAGTTTCTTAAAGAGTGTCACTTGAAGGCTCAATTGTCACTTATGACATTAGGAAGTGAAGGGACTCATCCATATGAGGCCCAAACTACCGTTTCTATCCTTATGCTTCATTCACCTAAAGGTAGTGAAAGCAGGTAGTGAACAAGGAATGTAACCAGGGGGTCCCCGGGGGATGTGCCCCCGNNNNNNNNNNNNNNNNNNNNNNNNNNNNNNNNNNNNNNNNNNNNNNNNNNNNNNNNNNNNNCCTGTAAATAGGCTTTCAAGGGAGTGAAGTGACTCATCAATATGAGGCACAAACGCTCTCACATGGACAAGCGACAGAAAGCAAGCCAGTATAGAGTTAAGAGCTTCACTCCTTAACCAAACTATCAGTAAACTAAGGAGCTCAACTGCTCTCAATCCCCCTGAGGGGGACTTAGGGTAACCTATGTTTTAAAAACCG | unknown | unknown | unknown | unknown | unknown |
| AL1-1 | 12 | 437832 | GCAGCTTGTACAAAAAAGTTTTGCTCGTTTGTTGTATGATGACTTCTTGAGAAATGCCAGGTTATTGTTTGTTTTCTTCATCCCTCTAGGAATTATGACATCTAGTTTTCAGAAGTTTTATCTACATTGTGCATGTACTTGGGGATTAATAATATCTGCTTCTATTTGCAGCTCCATTGATGAGGCTGCCAAAGAAAAGTTGGGACCATTTTCGACCTTATCTGGTGACGAAAGCTACAAGAGCCGGGATCTTGAAAAGGTATTCTAGAATTTTGAGAGTGATTATTATTAAGAGTGTTTTCTATTCGTTTACTTCCTTAAGATAATTTTGGATTATATCGTTCTTAAGATTGGACACTCTTCCTAATGATCATGTTGATCTAC | 100% | 1,00E-38 | 39% | XM_031091600.1 | Quercus lobata hydroxymethylglutaryl-CoA synthase (LOC115971599), mRNA |

**Table S6**: Environmental variables and their definitions used for GDM, LFMM, SFA, GLM and MLM genotype–environment association (GEA) analyses.

| **Database** | **Original name** | **Short name** | **Definition** |
| --- | --- | --- | --- |
| WorldClim 1.4 | prec5_16 | prec5_16 | average monthly precipitation (mm), May |
| WorldClim 1.4 | prec6_16 | prec6_16 | average monthly precipitation (mm), June |
| WorldClim 1.4 | bio3_16 | bio3_16 | Isothermality (BIO2/BIO7) (×100) |
| WorldClim 1.4 | bio8_16 | bio8_16 | Mean Temperature of Wettest Quarter |
| WorldClim 1.4 | bio15_16 | bio15_16 | Precipitation Seasonality (Coefficient of Variation) |
| WorldClim 1.4 | bio16_16 | bio16_16 | Precipitation of Wettest Quarter |
| WorldClim 1.4 | altitude | alt | altitude (elevation above sea level) (m) (from SRTM) |
| ENVIREM 1.0 | aridityIndexThornthwaite | aridity | Thornthwaite aridity index: Index of the degree of water deficit below water need |
| ENVIREM 1.0 | continentality | conti | continentality, the average temp. of warmest month - average temp. of coldest month (℃) |
| ENVIREM 1.0 | minTempWarmest | minTempWarm | min. temp. of the warmest month (℃ * 10) |
| ENVIREM 1.0 | monthCountByTemp10 | mCBTemp10 | count of the number of months with mean temp greater than 10℃ |
| ENVIREM 1.0 | PETColdestQuarter | PETCQ | mean monthly PET of coldest quarter (mm/month) |
| ENVIREM 1.0 | PETseasonality | PETseas | monthly variability in potential evapotranspiration (mm/month) |
| ENVIREM 1.0 | PETWettestQuarter | PETWetQ | mean monthly PET of wettest quarter (mm/month) |

**Table S7**: Summary of average adjusted *R*^2^, listed in Table 3, by each environmental predictor. Predictors are decreasingly ordered by the percentage of variation explained.

| **Predictor** | **50083_64** | **96506_153** | **96534_88** | **180975_104** | **227780_278** | **238771_249** | **277051_56** | **361438_297** | **437832_228** | **Sum** | **Variation explained (%)** |
| --- | --- | --- | --- | --- | --- | --- | --- | --- | --- | --- | --- |
| **aridity** | - | 0.108 | 0.116 | 0.095 | 0.128 | 0.045 | 0.076 | 0.109 | 0.140 | 0.817 | 81.7 |
| **PETCQ** | 0.107 | 0.076 | 0.110 | 0.101 | 0.166 | 0.070 | 0.103 | 0.066 | - | 0.799 | 79.9 |
| **bio8_16** | - | 0.062 | 0.081 | - | 0.115 | - | 0.112 | 0.080 | 0.069 | 0.519 | 51.9 |
| **prec6_16** | - | 0.055 | 0.062 | - | 0.127 | - | 0.043 | 0.099 | 0.111 | 0.497 | 49.7 |
| **PETWetQ** | - | - | 0.061 | - | 0.095 | - | 0.088 | 0.068 | 0.060 | 0.372 | 37.2 |
| **alt** | - | - | - | 0.064 | - | - | 0.060 | 0.073 | 0.071 | 0.268 | 26.8 |
| **PETseas** | 0.046 | - | - | - | - | 0.048 | - | 0.078 | - | 0.172 | 17.2 |
| **mCBTemp10** | 0.085 | - | - | - | - | 0.082 | - | - | - | 0.168 | 16.8 |
| **bio3_16** | 0.051 | - | - | - | 0.078 | - | - | - | - | 0.129 | 12.9 |
| **bio15_16** | 0.057 | - | - | - | - | 0.052 | - | - | - | 0.109 | 10.9 |
| **minTempWarm** | 0.032 | - | - | - | - | 0.024 | - | - | - | 0.055 | 5.5 |
| **prec5_16** | 0.045 | - | - | - | - | - | - | - | - | 0.045 | 4.5 |

**Figures**

**
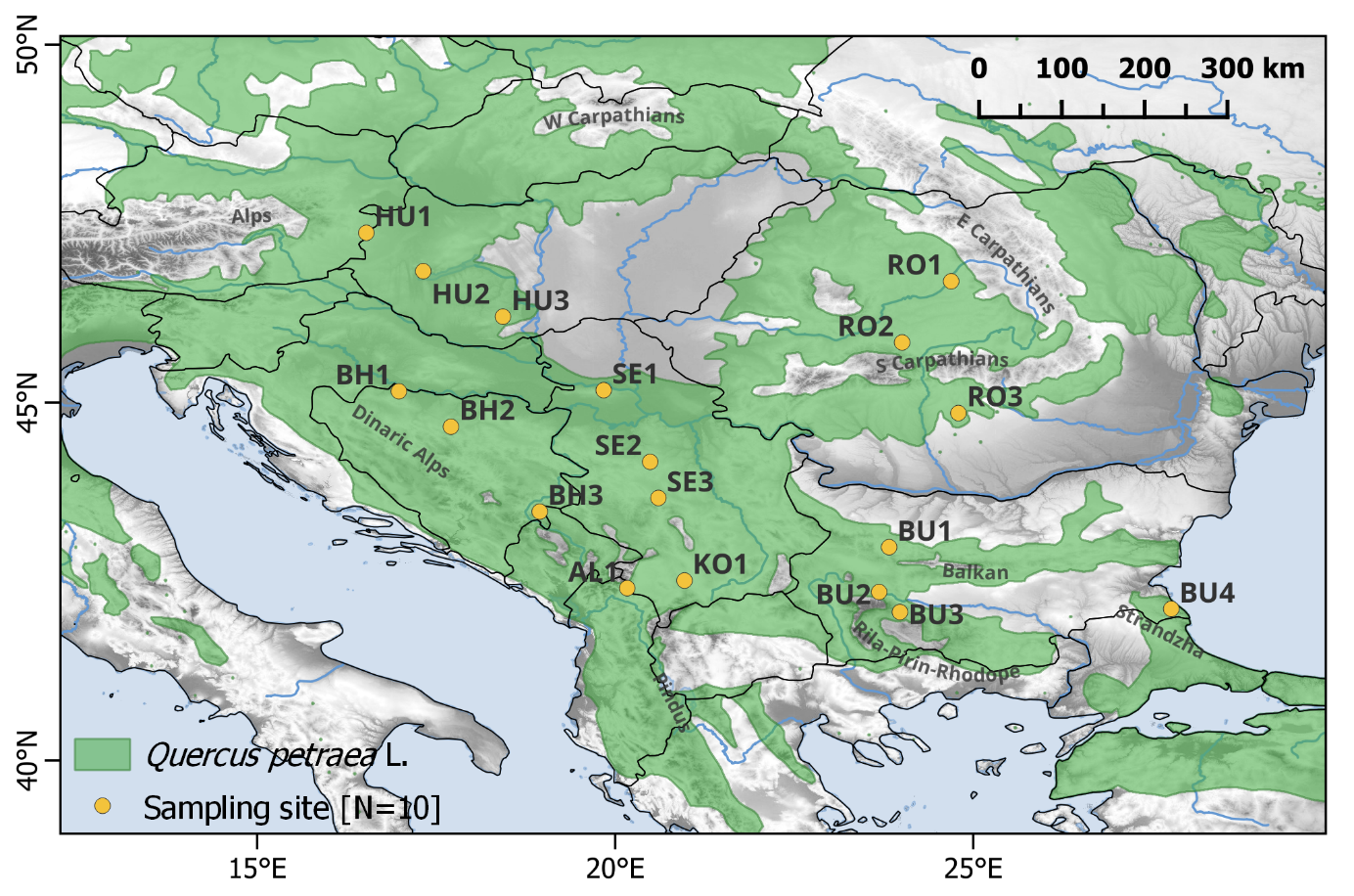
**

**Figure S1**: Distribution of *Quercus petraea* (L.) sampling locations across Europe (orange dots). Green areas represent the natural distribution of the species according to the EUFORGEN database (<http://www.euforgen.org/>)


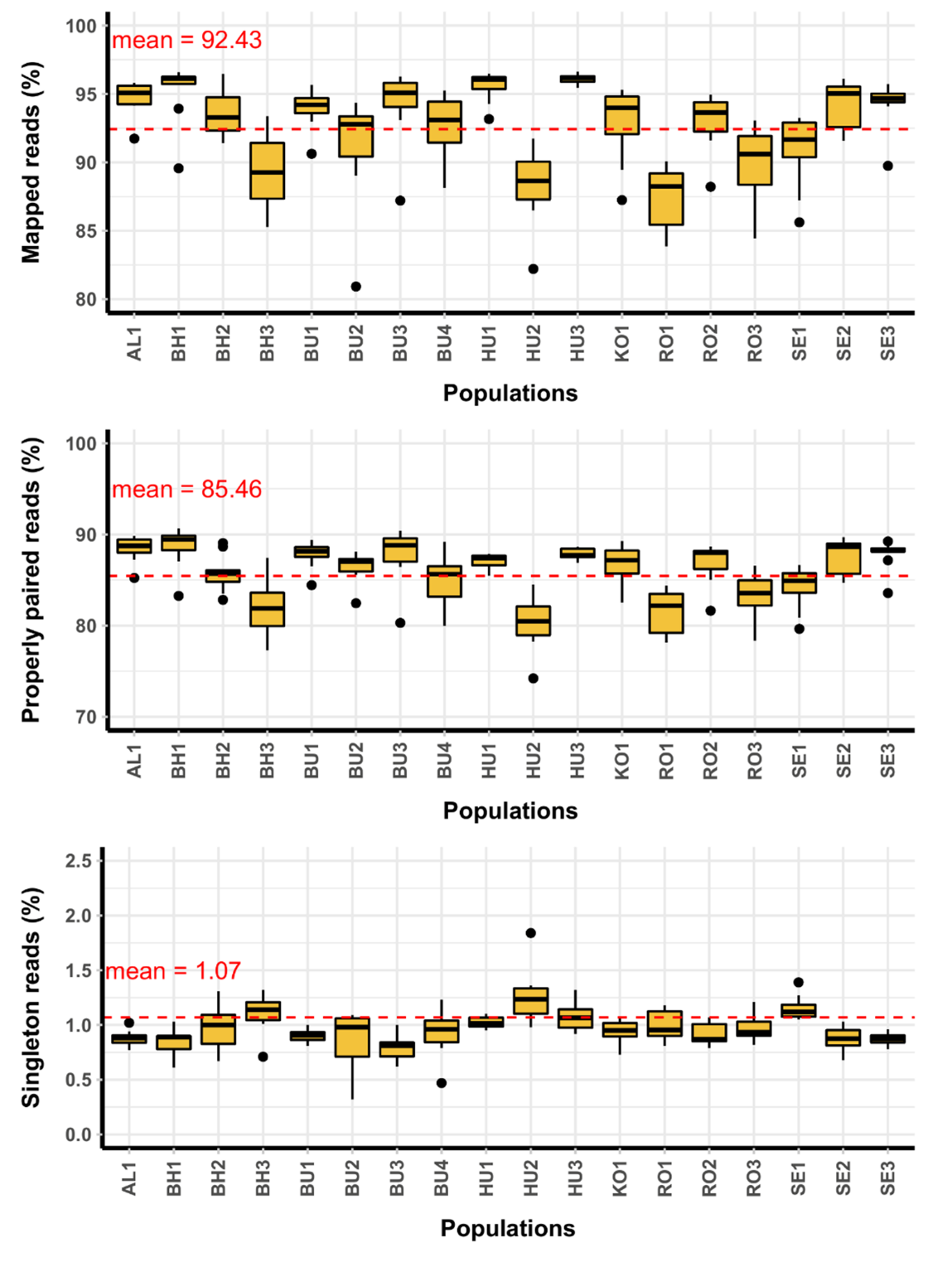


**Figure S2**: Mean percentage (%) of successfully mapped, paired and singleton reads by population. The dashed line represents the mean value of the populations.


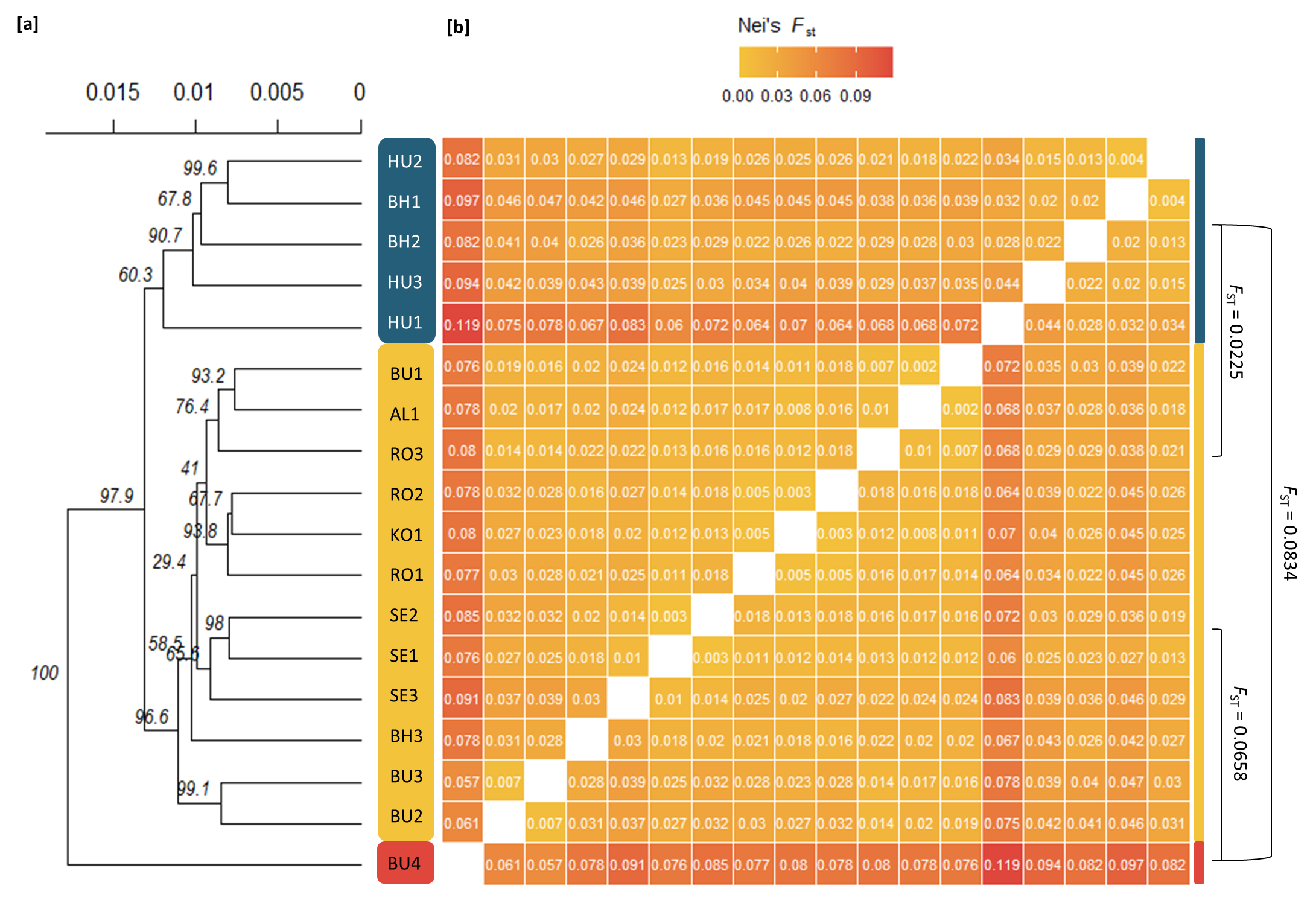


**Figure S3**: **a:** UPGMA dendrogram, based on the pairwise *F_ST_* values with 1000 bootstrap support, showing the genetic relatedness between the investigated populations. **b:** Heatmap of Nei's pairwise *F_ST_* calculated between all pairs of populations (Nei 1987). Darker colours indicate stronger differentiation. Population abbreviations are as explained in Tóth et al. 2021.
